# Physics-Based Synthetic Training for Automatic Target Detection in Radar Imaging

**DOI:** 10.3390/s26216875

**Published:** 2026-10-30

**Authors:** Lorena Pérez-Eijo, Marcos Arias, Jorge Martínez Fernández, Ignacio Sardinero-Meirás, Francisco N. Pérez Fernández, Borja Gonzalez-Valdes, Yolanda Rodríguez-Vaqueiro, José Vázquez-Cabo, Jesús Grajal

**Affiliations:** 1 AtlanTTic Research Center, Universidade de Vigo, 36310 Vigo, Spain; marcos@com.uvigo.es (M.A.); jorgemtezfer@gmail.com (J.M.F.); bgvaldes@com.uvigo.es (B.G.-V.); yrvaqueiro@com.uvigo.es (Y.R.-V.); jvcabo@com.uvigo.es (J.V.-C.); 2 Information Processing and Telecommunications Center, Universidad Politécnica de Madrid, 28040 Madrid, Spain; i.sardinero@upm.es (I.S.-M.); fn.perez@upm.es (F.N.P.F.); jesus.grajal@upm.es (J.G.)

**Keywords:** D-band radar imaging, electromagnetic simulation, physical optics, automatic target detection, synthetic training data, simulation-to-measurement transfer, near-field radar

## Abstract

Representative labeled radar data are difficult to acquire across the target and scene variability needed for automatic detection. Physics-based simulation offers an alternative, but its value depends on whether detectors trained on simulated responses generalize beyond training targets. This work examines that question using acquisition-aware electromagnetic simulation of a D-band multiple-input multiple-output (MIMO) radar. Complex multistatic responses are generated before image formation, and simulated and measured data undergo the same coherent reconstruction and detector-input preparation. No measured target responses are used during development. Seven convolutional neural network (CNN) detectors are compared, with YOLOX-Tiny selected based on detection performance and inference cost. Across seven geometry-disjoint folds, YOLOX-Tiny achieves mean $F_{2}=0.89$ and ${\mathrm{mAP}}_{0.5}=0.88$ for single-object scenes, and $F_{2}=0.82$ and ${\mathrm{mAP}}_{0.5}=0.79$ for multi-object scenes. Generalization is further examined on 14 independent jointly simulated multi-object scenes and measurements from the reference D-band radar and two additional systems. The measurements provide qualitative evidence of transfer and reveal replica-related false detections and local class ambiguities in high-resolution responses. Lower-resolution measurements retain correctly detected targets alongside additional false detections assigned to other classes. These findings highlight the need for more representative synthetic radar responses and for relating image detections to physical targets.

## 1. Introduction

High-resolution near-field millimeter-wave and sub-terahertz radar imaging provides non-ionizing sensing capabilities for security screening, industrial inspection, and concealed-object detection [1,2]. Learning-based interpretation requires representative labeled data, but acquiring measurements across target geometries, poses, and scene configurations is costly. Physics-based simulation offers an alternative. Its usefulness for detector training depends on whether the learned features remain informative beyond the target models and acquisition conditions represented during development.

Learning-based detection, localization, and recognition have been demonstrated on measured millimeter-wave and sub-terahertz radar images [3,4,5,6]. Simulation-based training has also been investigated for concealed-object segmentation using full-wave electromagnetic (EM) models [7] and for YOLO-based detection using EM-scattering-based images [8]. Studies of simulation-to-measurement transfer differ in their tasks and use of measured information. Physical-optics target responses combined with measured backgrounds have supported radar classification [9], while synthetic target signatures embedded in measured SAR backgrounds have supported object detection [10]. RadSimReal and the indoor radar framework of Reitz et al. evaluate models trained on synthetic data using measured observations [11,12]. Recent SAR recognition studies additionally address generalization from fully simulated training data [13] and adaptation using unlabeled measured data [14].

This work examines two distinct questions: generalization to unseen target geometries and transfer to measured radar data.

**Figure 1:**
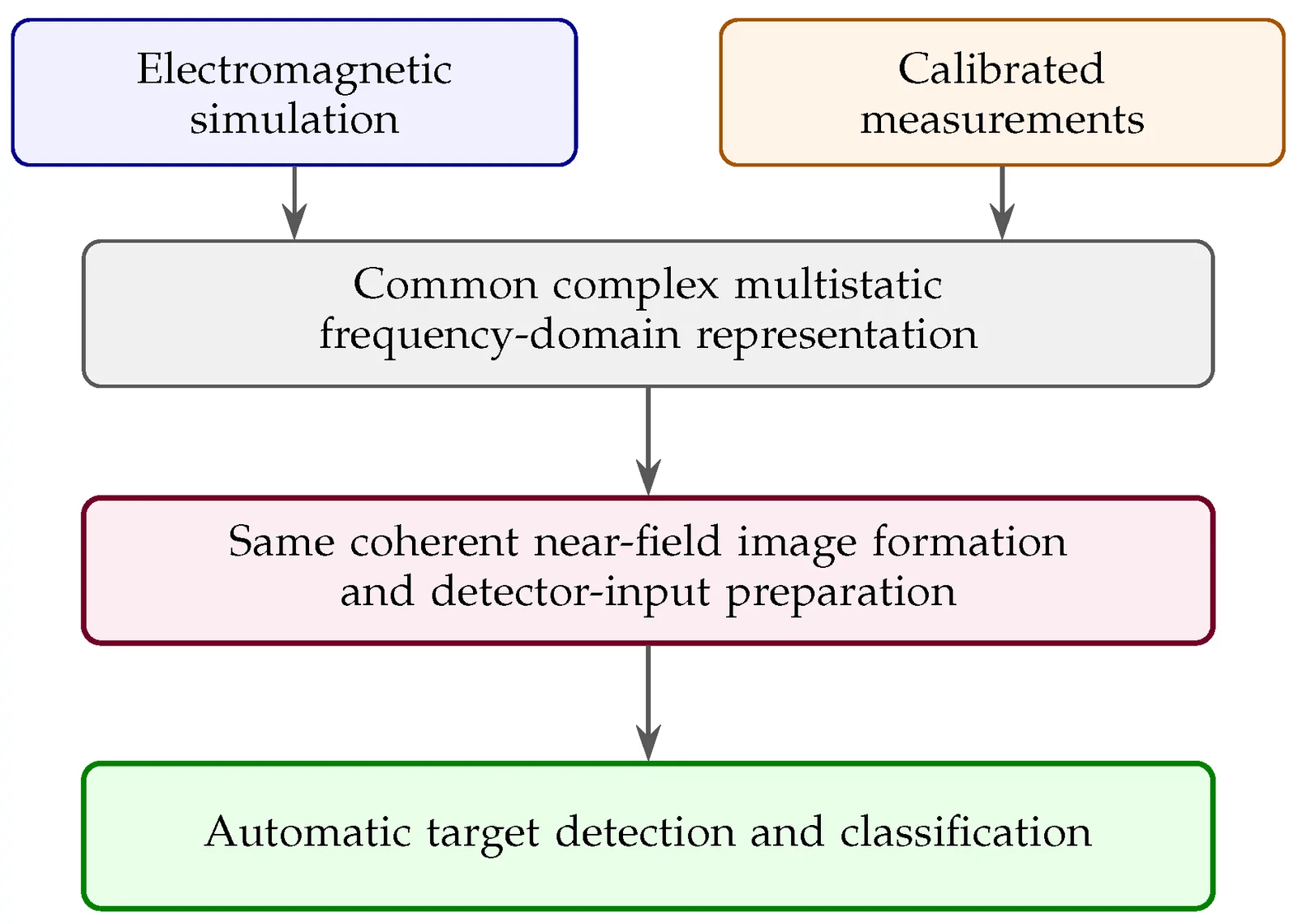
Common processing chain for simulated and measured radar responses. Only simulated target responses are used for detector development; measured target responses are reserved for qualitative evaluation.

A coherent radar image depends on both target scattering and how the scattered field is sampled and reconstructed. Frequency sampling, transmitter–receiver geometry, and aperture extent shape the response presented to the detector [15,16,17]. Recent near-field multiple-input multiple-output (MIMO) simulation research further demonstrates the importance of polarization and multiple-bounce propagation when these mechanisms contribute substantially to the measured response [18]. Here, the physical-optics (PO) simulator described in [19] generates complex multistatic responses using the frequency sampling, array geometry, and mechanical scan of an experimental D-band MIMO radar [20]. Simulated responses and calibrated measurements undergo the same coherent near-field reconstruction and detector-input preparation, with reconstruction using the acquisition geometry of each radar, as summarized in Figure 1.

All target responses used for detector development are simulated for the reference D-band configuration. Measured target responses are reserved for evaluation, while class-independent statistics derived from measured backgrounds support background augmentation. Seven detector configurations are compared, and the selected architecture is retrained using the synthetic development dataset. The evaluation addresses three objectives:

1. To assess generalization to knife and pistol geometries excluded from model development.
2. To quantify final-model performance on independent multi-object scenes simulated jointly, with all targets contributing to the same complex multistatic response before reconstruction.
3. To examine qualitative transfer and failure modes without detector adaptation using measurements from the reference D-band radar, a later-generation D-band array, and a lower-resolution K-band system.

The measured cases provide diagnostic evidence of transfer, rather than a statistical assessment of cross-system performance.

Section 2 describes the radar acquisition and EM simulation, Section 3 presents image formation, and Section 4 describes detector development. Section 5 defines the evaluation protocol. Section 6 presents the results and discussion, followed by the conclusions in Section 7.

## 2. D-Band MIMO Radar Acquisition and Electromagnetic Simulation

This section describes the D-band radar configuration used to generate the synthetic training data and acquire the corresponding same-system measurements. The radar employs a linear frequency-modulated continuous-wave (LFMCW) waveform spanning 118 to 133.6 GHz and combines a sparse linear MIMO array with a mechanically synthesized vertical aperture. Eight transmitters (Tx) and seven receivers (Rx) provide 56 bistatic channels. The array lies along the *x*-axis, the *y*-axis defines range, and translation of the radar head along the *z*-axis synthesizes the vertical aperture for imaging in an XZ plane. The hardware, calibration procedure, and experimental characterization have been reported previously [20].

**Figure 2:**
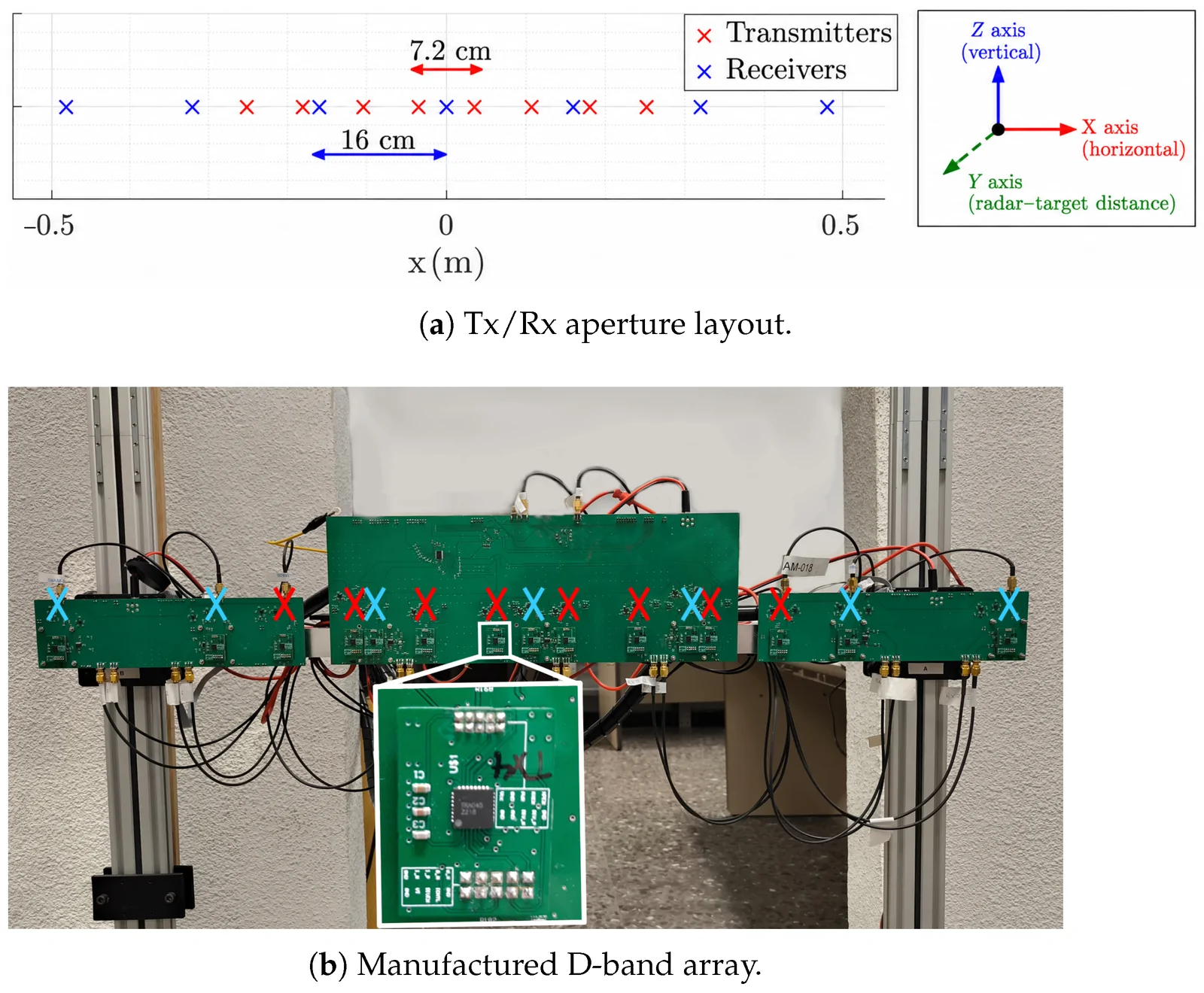
D -band MIMO radar aperture. (**a**) Sparse linear Tx/Rx layout along the *x*-axis, with mechanical scanning along *z* and range along *y*, normal to the XZ plane. (**b**) Manufactured array used for the experimental acquisitions. The color coding in (**b**) follows the legend in (**a**): red crosses indicate transmitters, whereas blue crosses indicate receivers.

Figure 2 shows the Tx/Rx layout and the manufactured array. Table 1 lists the acquisition parameters shared by the simulation and this radar.

### 2.1. Electromagnetic Simulation

Synthetic complex multistatic responses are generated using the PO simulator described in [19]. For each combination of radar-head position, transmitter, receiver, and frequency sample, the simulator computes the complex scattered field at the receiver. The frequency sampling, Tx/Rx geometry, and mechanical scan follow the parameters listed in Table 1.

The simulated targets are modeled as perfectly electrically conducting (PEC) surfaces and discretized using triangular meshes, with surface coordinates denoted by $r_{s}$. This material assumption applies to the synthetic targets; the framework could accommodate other materials through an EM solver that provides their complex scattered responses. The maximum mesh-edge length is $\lambda_{\min}/2$, where $\lambda_{\min}$ is the wavelength at the highest operating frequency. For $f_{\max}=133.6\;\mathrm{GHz}$, this corresponds to approximately 1.12 mm.

For each transmitter and frequency sample, the incident field is evaluated over the target mesh. On each locally planar facet illuminated by the transmitter, the induced current is approximated by the PO current

(1) $$J_{\mathrm{PO}}\left(r_{s}\right)≃2\;\hat{n}\left(r_{s}\right)\times H^{\mathrm{inc}}\left(r_{s}\right),$$

where $\hat{n}\left(r_{s}\right)$ is the outward unit normal and $H^{\mathrm{inc}}\left(r_{s}\right)$ is the incident magnetic field.

The field radiated by the PO currents is evaluated at each receiver, including contributions only from illuminated facets visible from that receiver. Propagation uses the corresponding near-field Tx–facet and facet–Rx distances, preserving the complex frequency-dependent phase of each bistatic path. The adopted PO model does not explicitly include multiple scattering between target facets or edge diffraction.

The resulting complex bistatic frequency-domain responses are arranged as

(2) $$D^{\mathrm{sim}}=\left\{S_{q,n}^{\mathrm{sim}}\left(f_{k}\right)\right\},$$

where $k=1,\ldots ,N_{f}$ indexes the $N_{f}=$ 10,000 frequency samples, $n=1,\ldots ,N_{z}$ indexes the $N_{z}=126$ vertical scan positions, and q≡(m,𝓁) identifies one of the $N_{q}=56$ bistatic channels formed by transmitter *m* and receiver *ℓ*. At each scan position, the transmitter and receiver coordinates, $r_{m,n}^{\mathrm{Tx}}$ and $r_{𝓁,n}^{\mathrm{Rx}}$, follow the translated radar-head geometry.

### 2.2. Experimental Acquisition

Measurements are acquired using the radar configuration summarized in Table 1. At each vertical scan position, the transmitters are activated sequentially, and the complex frequency-domain responses are recorded across all receiver channels. Calibration and trace conditioning follow [20] and include channel-dependent phase and delay corrections, together with the range alignment required for coherent multistatic image formation.

Let $\Delta z_{n}$ denote the vertical displacement of the radar head at the *n*-th scan position, with $\Delta z_{n}\in \left[-0.35,\;0.35\right]\;m$ and a sampling step of 5.6mm. The transmitter and receiver coordinates are therefore

(3) $$r_{m,n}^{\mathrm{Tx}}=r_{m,0}^{\mathrm{Tx}}+\Delta z_{n}\hat{z},\;r_{𝓁,n}^{\mathrm{Rx}}=r_{𝓁,0}^{\mathrm{Rx}}+\Delta z_{n}\hat{z},$$

where the subscript 0 denotes the reference radar-head position.

Using the same indexing introduced in (2), the measured responses are arranged as

(4) $$D^{\mathrm{meas}}=\left\{S_{q,n}^{\mathrm{meas}}\left(f_{k}\right)\right\},$$

where $S_{q,n}^{\mathrm{meas}}\left(f_{k}\right)$ denotes the calibrated and trace-conditioned complex response for bistatic channel *q*, scan position *n*, and frequency sample $f_{k}$.

For this D-band configuration, simulated and calibrated measured responses share the same channel indexing, frequency samples, and translated-aperture geometry. Both are organized as a 56 × 126 × 10,000 data cube, with dimensions corresponding to bistatic channels, vertical scan positions, and frequency samples, respectively. They are processed using the same image-formation operator, without spatial or frequency resampling.

## 3. Coherent Near-Field Image Formation

Radar images are formed by coherent near-field bistatic backprojection. The coarse-to-fine procedure described below reduces computational cost by restricting fine-grid reconstruction to selected regions, without changing the underlying complex responses or the reconstruction operator.

To simplify the notation, the composite index a≡(q,n), with q≡(m,𝓁), groups the bistatic-channel and vertical-scan indices. Here, ${\tilde{S}}_{a}\left(f_{k}\right)$ denotes the complex frequency response, and $r_{a}^{\mathrm{Tx}}$ and $r_{a}^{\mathrm{Rx}}$ are the corresponding transmitter and receiver positions.

### 3.1. Coherent Near-Field Bistatic Backprojection

The radar image is reconstructed on the XZ plane located at the fixed range $Y_{\mathrm{img}}$. A generic image point is

(5) $$r_{XZ}\left(x,z\right)={\left[x,\;Y_{\mathrm{img}},\;z\right]}^{T}.$$

The Cartesian reconstruction grid is denoted by

(6) $$P=\left\{\left(x_{i},z_{j}\right):i=1,\ldots ,N_{x}^{\mathrm{img}},\;j=1,\ldots ,N_{z}^{\mathrm{img}}\right\},$$

where $N_{x}^{\mathrm{img}}$ and $N_{z}^{\mathrm{img}}$ are the numbers of evaluated image points along the *x*- and *z*-directions, respectively.

For acquisition *a*, the bistatic propagation distance associated with an image point is

(7) $$R_{a}\left(x,z\right)=∥r_{XZ}\left(x,z\right)-r_{a}^{\mathrm{Tx}}∥+∥r_{XZ}\left(x,z\right)-r_{a}^{\mathrm{Rx}}∥.$$

The full bistatic propagation path is retained because the acquisition operates in the near field. Coherent bistatic backprojection compensates the propagation phase associated with each candidate image point and coherently combines the frequency and multistatic measurements [15,20,21]:

(8) $$I\left(x,z\right)=\sum_{a=1}^{N_{a}}\sum_{k=1}^{N_{f}}{\tilde{S}}_{a}\left(f_{k}\right)\;e^{jk_{0}\left(f_{k}\right)R_{a}\left(x,z\right)},\;k_{0}\left(f_{k}\right)=\frac{2\pi f_{k}}{c_{0}},$$

where $N_{a}=N_{q}N_{z}$ is the number of bistatic-channel and scan-position combinations, and $N_{f}$ is the number of frequency samples. For the reference D-band configuration, $N_{a}=56\times 126=7056$ and $N_{f}=$ 10,000.

With the phasor convention adopted here, propagation over the bistatic path $R_{a}$ contributes the phase factor $\exp[-jk_{0}\left(f_{k}\right)R_{a}]$ to ${\tilde{S}}_{a}\left(f_{k}\right)$. Backprojection therefore applies the conjugate phase factor in (8), so that contributions associated with the correct image position add coherently.

For any reconstruction grid P, the magnitude image is peak-normalized according to

(9) $$D\left(x,z\right)=\frac{\left|I(x,z)\right|}{\max_{(x^{\prime },z^{\prime })\in P}\left|I\left(x^{\prime },z^{\prime }\right)\right|}.$$

The same backprojection and normalization operations are applied to simulated and measured responses.

### 3.2. Coarse-to-Fine Local Reconstruction

A coarse reconstruction of the complete inspection plane identifies regions of interest (ROIs) for fine-grid reconstruction. Backprojection is then recomputed within these regions directly from the original complex multistatic responses. Only the resulting fine-grid images are passed to the detector.

The coarse Cartesian grid uses

(10) $$\Delta x_{\mathrm{coarse}}=\Delta z_{\mathrm{coarse}}=5\lambda_{\min},\;\lambda_{\min}=\frac{c_{0}}{f_{\max}}.$$

For $f_{\max}=133.6\;\mathrm{GHz}$, the sampling interval is approximately 11.2mm. The inspection plane spans x,z∈[−0.5,0.5]m, giving a 1m×1m area with approximately 90×90 grid points. The coarse spacing is five times the fine spacing in each direction, reducing the number of points per unit area by approximately a factor of 25.

ROI selection follows a deterministic procedure. The peak-normalized coarse magnitude image, $D^{\mathrm{coarse}}\left(x,z\right)$, is thresholded in linear scale using Otsu’s criterion [22]. Eight-connected components are extracted from the binary mask, and each component is enclosed by an axis-aligned bounding box.

Each candidate box is expanded by one coarse-grid pixel in all four directions and rasterized onto an auxiliary binary mask. A second eight-connected-component analysis groups expanded boxes that touch or overlap. The bounding box of each group is then expanded by one additional coarse-grid pixel in all four directions, subject to the inspection-plane boundaries. These expansions provide a margin against coarse-grid quantization and fragmentation of the thresholded response. Grouping nearby responses allows them to be reconstructed within a single ROI, reducing repeated setup and data-access overhead from separate fine-reconstruction calls.

For each resulting ROI $P_{i}$, backprojection in (8) is evaluated on a grid with spacing

(11) $$\Delta x_{\mathrm{fine}}=\Delta z_{\mathrm{fine}}=\lambda_{\min}≃2.24\;\mathrm{mm}.$$

Each fine-grid image is reconstructed over the entire selected ROI, rather than obtained by cropping or interpolating the coarse image. The reconstruction therefore includes responses within the ROI that did not exceed the coarse threshold, including localized secondary responses produced by the sidelobe structure of the imaging system’s point-spread function (PSF). Such non-physical secondary responses are referred to here as replicas [15].

The magnitude image is peak-normalized over $P_{i}$ to obtain $D_{i}^{\mathrm{fine}}\left(x,z\right)$. The grid spacing determines numerical sampling, not physical resolution, which depends on the radar bandwidth, aperture, acquisition geometry, and focusing operator.

**Figure 3:**
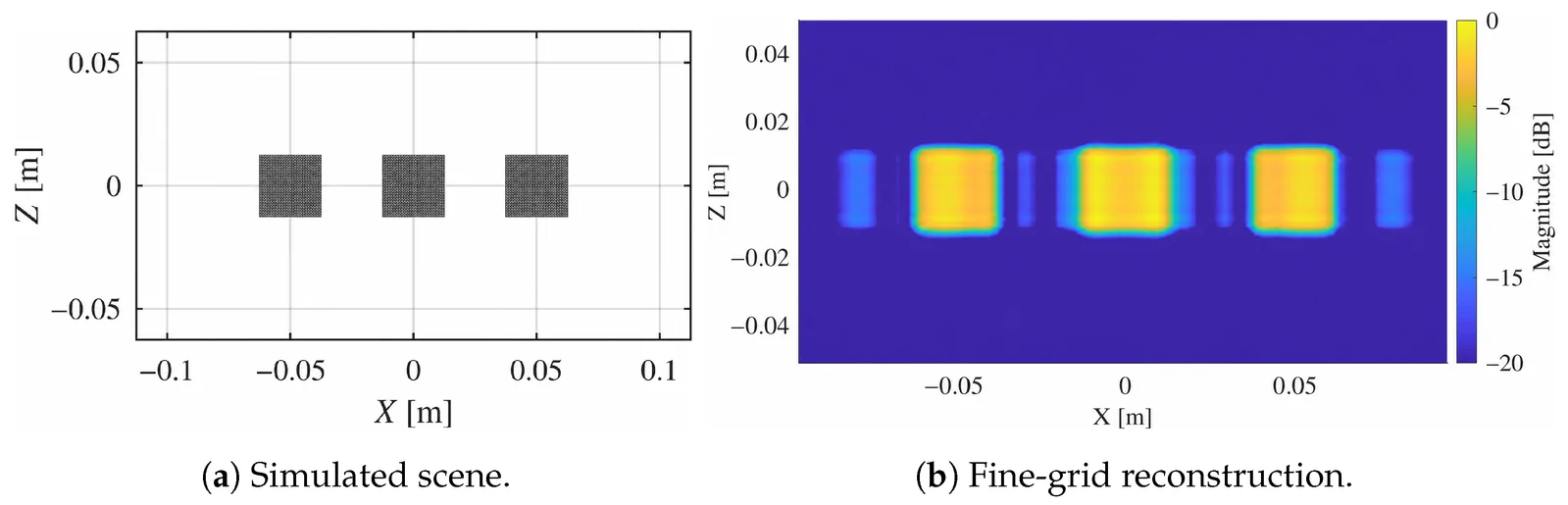
Simulated three-plate scene. (**a**) Target geometry. (**b**) Fine-grid coherent reconstruction within the selected ROI.

**Figure 4:**
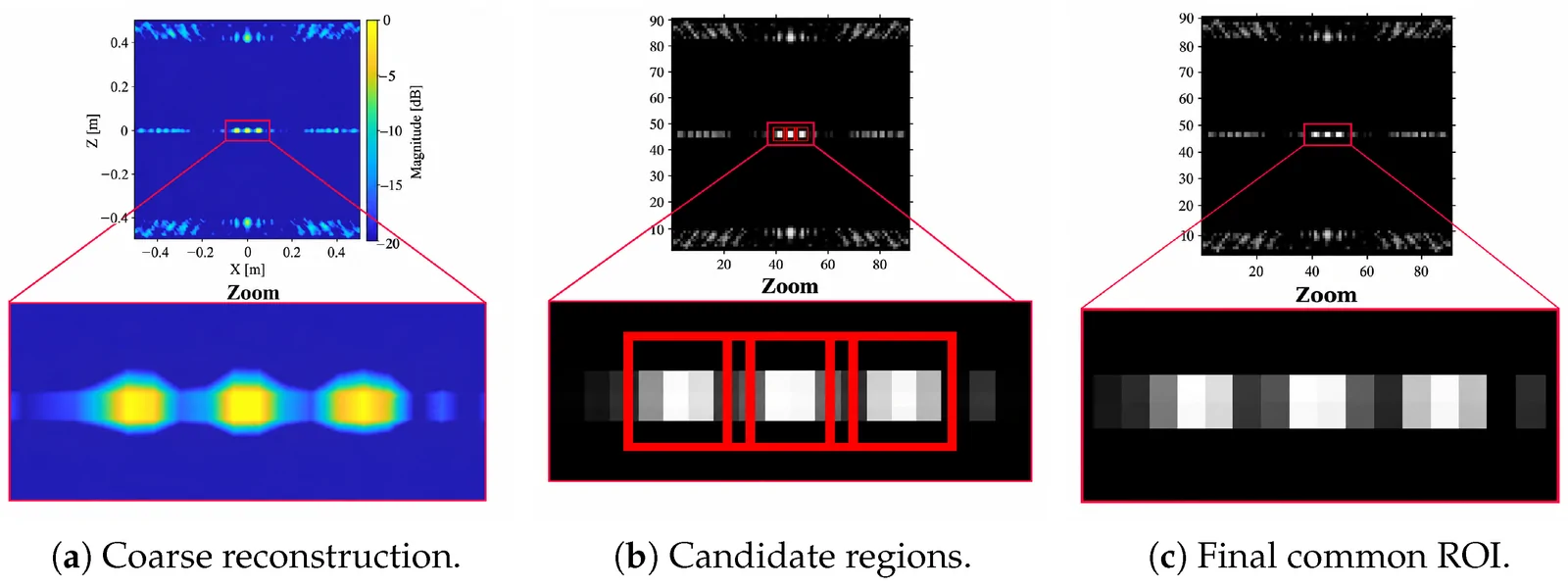
ROI selection for the simulated three-plate scene. (**a**) Detail of the coarse reconstruction of the full inspection plane. (**b**) Candidate regions after Otsu thresholding and connected-component analysis. (**c**) Common ROI after box expansion and grouping.

Figure 3 and Figure 4 illustrate the final reconstruction and intermediate ROI-selection stages for a simulated three-plate scene.

The same reconstruction and ROI-selection procedure is applied to all simulated and measured data. The resulting $D_{i}^{\mathrm{fine}}\left(x,z\right)$ images are converted into the detector input representation described in the following section.

## 4. Simulation-Based Detector Development

This section describes the detector input representation, the synthetic development dataset, the training protocol, and the detection metrics. Detector development relies exclusively on simulated target responses generated for the D-band radar described in Section 2.

### 4.1. Detector Input Representation

The detector operates on the fine-grid radar reconstruction described in Section 3. For each reconstructed region of interest (ROI), the peak-normalized magnitude image is converted to decibels, clipped to [−20,0]dB, and linearly mapped to [0,1].

The resulting image is quantized to 8 bits and placed on a fixed 224×224-pixel canvas without spatial resampling. Smaller ROIs are symmetrically zero-padded, whereas larger ROIs are centrally cropped. This choice standardizes the detector input size while preserving the native spatial sampling of each radar reconstruction, avoiding interpolation-induced changes in target morphology and apparent target size. Thus, the 224×224 canvas fixes only the detector input dimensions; the spatial sampling interval remains that of each radar reconstruction and may therefore differ across radar systems.

Because the radar representation contains a single intensity channel, the same grayscale image is copied identically into the three input channels expected by the detector implementations. This is performed solely for architectural compatibility; no color information or additional image content is introduced.

The same detector-input preparation is applied to all simulated and measured radar images.

### 4.2. Synthetic Radar Dataset

The detector dataset is generated from the PO-simulated complex responses described in Section 2.1, using the reconstruction and detector-input preparation defined above.

**Figure 5:**
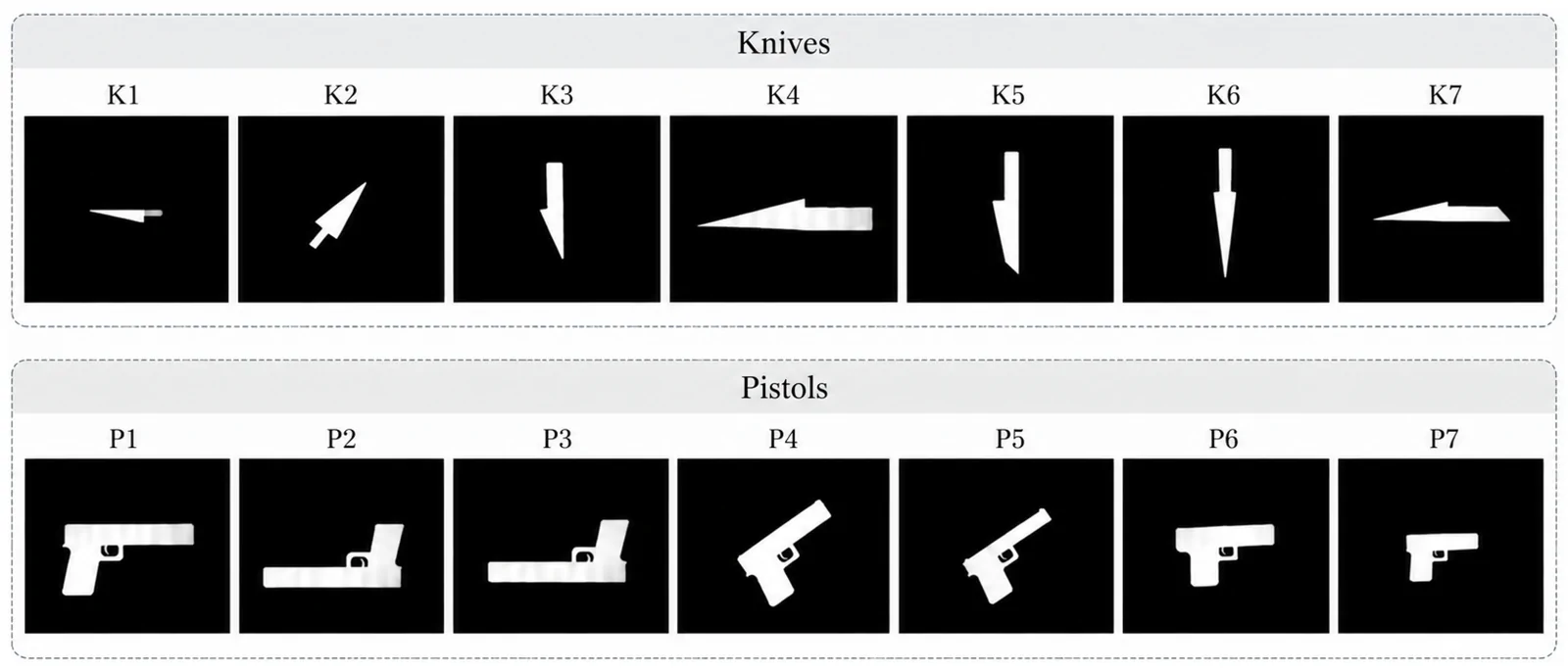
Target geometries used to generate the synthetic radar dataset. K1–K7 denote the seven knife geometries and P1–P7 the seven pistol geometries.

Three target classes are considered: knife, pistol, and square metallic plate. The knife and pistol classes each contain seven simplified planar geometries, denoted K1–K7 and P1–P7, respectively, and shown in Figure 5. The square-plate class contains 13 side lengths ranging from 3 to 15cm.

Targets are placed at a nominal range of 2m, with their *x*- and *z*-coordinates varied within [−0.20,0.20]m. To broaden the range of physical configurations represented in the synthetic dataset, selected targets are also rotated within the imaging plane, with angles ranging from 0 to 360 degrees. Each configuration is simulated at the electromagnetic-response level, where translations modify the near-field Tx–target–Rx propagation paths and viewing geometry, while rotations change the orientation of the target’s scattering features relative to the fixed radar aperture. These variations are therefore captured in the complex multistatic responses used for image formation. Positional diversity is not introduced by translating reconstructed images.

The single-object dataset contains 383 radar images, distributed as summarized in Table 2.

The development pool also includes 29 jointly simulated multi-object scenes. Each scene is generated as a single electromagnetic acquisition, with all targets contributing coherently to the same multistatic response before reconstruction. Together, the 383 single-object images and 29 multi-object scenes form a development pool of 412 radar images containing 454 annotated target instances.

### 4.3. Detector Architectures and Training Protocol

Seven detector configurations are evaluated: YOLOX-Tiny and YOLOX-S [23], YOLOv4-Tiny [24], SSD-MobileNet and SSD-ResNet50 [25], and Faster R-CNN-ResNet18 and Faster R-CNN-MobileNet [26].

The benchmark covers different detection paradigms. YOLOX, YOLOv4, and SSD are one-stage detectors, whereas Faster R-CNN follows a two-stage region-proposal and classification-and-refinement pipeline. Both anchor-based and anchor-free architectures are included.

All detectors use the same 224×224-pixel radar representation, target classes, annotations, and data-handling protocol. Training and inference are implemented in MATLAB R2025b using the Deep Learning Toolbox and Computer Vision Toolbox. Each network is initialized from pretrained weights, with its final detection layers adapted to the three target classes.

Ground-truth bounding boxes and class labels are manually annotated on the final 224-by-224-pixel radar images using MATLAB Image Labeler. The annotations therefore correspond to the reconstructed radar responses rather than being derived directly from the target geometries.

For every training run, the pseudorandom generator is initialized with seed 0. The model-development data are split image-wise in a 70:15:15 ratio into training, validation, and internal-development subsets. Validation is used for early stopping and checkpoint selection, whereas the internal-development subset is used only for qualitative verification and does not contribute to the reported metrics.

During detector training, online image-domain augmentation is applied only after data partitioning and exclusively to the training subset. It includes rotations over the full angular range with $1^{\circ }$ resolution, reflections about the image axes, uniform scale factors in [0.5,1.25], and multiplicative amplitude factors in [0.7,1.2]. These transformations are intended to improve detector robustness rather than reproduce additional electromagnetic acquisitions. Unlike physically rotating a target relative to the fixed radar aperture, rotating a reconstructed image also rotates its associated sidelobe and replica structure.

As part of the training augmentation, background variability is introduced using class-independent noise models fitted to measured radar backgrounds. For each augmented image, a random background realization is drawn from either a Weibull or a Gamma distribution. For the Weibull model, the shape and scale parameters are sampled from [1.57,1.90] and [0.10,0.19], respectively. For the Gamma model, the corresponding ranges are [2.18,2.80] and [0.04,0.06]. The generated background is then processed using the same logarithmic mapping and dynamic-range clipping defined in Section 4.1. It is combined with the simulated image by retaining the larger value at each pixel. This adds measured-like low-level background structure while preserving the dominant simulated target response. No class weighting or class-balanced sampling was applied. Online image-domain augmentation was intended to increase training variability and detector robustness, rather than to compensate for differences in class frequencies.

For anchor-based detectors, six anchors are estimated from the corresponding training-set bounding boxes using *k*-means clustering with 1−IoU as the distance measure. Anchor estimation is performed using MATLAB’s estimateAnchorBoxes function and repeated independently for each geometry-disjoint fold using only its training annotations. YOLOv4-Tiny and SSD use two groups of three anchors, whereas Faster R-CNN uses all six. YOLOX-Tiny and YOLOX-S are anchor-free and therefore do not require anchor estimation.

YOLO and SSD models are optimized with Adam [27], whereas Faster R-CNN models use stochastic gradient descent with momentum 0.9. All architectures use an initial learning rate of $10^{-3}$, reduced by a factor of 0.5 every 50 epochs. Batch sizes are 16 for the one-stage detectors and 4 for Faster R-CNN.

Validation loss is evaluated every 25 iterations. Early stopping [28] is triggered after 50 consecutive validation checks without improvement for YOLO and SSD and after 200 checks for Faster R-CNN, corresponding in both cases to an effective patience of 20,000 processed training samples. Training is capped at 400 epochs, and the checkpoint with the lowest validation loss is retained.

### 4.4. Detection Metrics

Detector performance is evaluated using the $F_{2}$ score and mean average precision at an intersection-over-union (IoU) threshold of 0.5 (${\mathrm{mAP}}_{0.5}$). The $F_{2}$ score evaluates a fixed operating point, whereas ${\mathrm{mAP}}_{0.5}$ summarizes performance as the confidence threshold is varied.

Nonmaximum suppression (NMS) is handled internally by MATLAB’s detect function using the default settings for each architecture. YOLOX uses an IoU overlap threshold of 0.45, while YOLOv4 and SSD use 0.5. Faster R-CNN uses a threshold of 0.5, with overlap defined as the intersection area divided by the area of the smaller bounding box rather than the union area. These settings are kept unchanged throughout the evaluation.

The $F_{2}$ score is defined as

(12) $$F_{2}=5\frac{\mathrm{Precision}\;\mathrm{Recall}}{4\;\mathrm{Precision}+\mathrm{Recall}},$$

and gives greater weight to recall than to precision. This is appropriate for the screening-oriented application considered here, where missing a target is more detrimental than generating an additional detection.

For the precision–recall analysis, detections are retrieved using a minimum confidence threshold of 0.001. For $F_{2}$, detections with confidence below 0.5 are discarded, and the remaining predictions are matched one-to-one with ground-truth boxes. A prediction is counted as a true positive when its predicted class is correct and its overlap with an unmatched ground-truth box satisfies IoU≥0.5 [29,30]. Unmatched ground-truth targets are counted as false negatives, whereas wrong-class predictions, duplicate detections, and predictions with insufficient overlap are counted as false positives.

Average precision (AP) is computed from the class-specific precision–recall curves at an IoU threshold of 0.5. For each class, AP is obtained as the absolute value of the trapezoidal integral of precision with respect to recall, using MATLAB’s trapz function without additional precision-envelope interpolation. The class-specific AP values are averaged to obtain ${\mathrm{mAP}}_{0.5}$.

For quantitative evaluation on synthetic radar data, each held-out image is evaluated under 216 image-domain transformations: 12 in-plane rotations from 0 to 330 degrees in 30-degree increments, three scale factors (0.75, 1.0, 1.25), two reflection conditions (original and horizontally flipped), and three background conditions (no additional noise, Weibull, and Gamma). No additional amplitude scaling is applied. Unlike training background augmentation, evaluation noise is added to the transformed images.

The corresponding ground-truth boxes undergo the same geometric transformations and are clipped to the image boundaries, with boxes discarded when they do not satisfy the transformation criteria. The detector is applied independently to each transformed image. Predictions and transformed annotations are pooled before computing the class-specific precision–recall curves and detection metrics.

These transformations do not represent additional electromagnetic acquisitions or independent target geometries. The reported quantitative metrics therefore characterize detector performance over the transformed test images rather than exclusively over the original radar reconstructions.

Inference time is measured for a single 224×224-pixel input with batch size one, from detector invocation until its output is returned, using an Intel Core i9-13900K CPU (Intel Corporation, Santa Clara, CA, USA) and an NVIDIA GeForce RTX 4090 GPU (NVIDIA Corporation, Santa Clara, CA, USA). The reported time includes detector inference only and excludes radar acquisition, image formation, ROI selection, and detector-input preparation.

## 5. Experimental Setup

The evaluation comprises a geometry-disjoint architecture benchmark, independent multi-object testing, and a qualitative assessment using measured radar data.

### 5.1. Geometry-Disjoint Architecture Benchmark

The architecture benchmark evaluates whether detectors trained on a given set of target geometries generalize to knife and pistol geometries not represented during model development. A random image-level split would not provide this separation because images acquired at different positions of the same geometry may retain geometry-specific scattering characteristics.

A seven-fold geometry-disjoint protocol is therefore defined from the knife and pistol geometries introduced in Section 4.2. In fold *r*, knife geometry K_*r*_ and pistol geometry P_*r*_, r=1,…,7, are excluded entirely from model development. All single-object images generated from either geometry are removed, together with any of the 29 multi-object scenes containing K_*r*_, P_*r*_, or both.

The geometry-based exclusion is performed before data partitioning and before any image-domain augmentation. The remaining images are then split according to the 70:15:15 training, validation, and internal-development protocol described in Section 4.3, with training augmentation restricted to the training subset. The excluded images remain outside model development and are subjected to the transformed-test evaluation protocol described in Section 4.4.

The geometry-disjoint exclusion applies only to the knife and pistol classes. The square metallic plate class remains represented during model development in every fold and may also occur in the held-out multi-object scenes. The reported benchmark averages are restricted to the knife and pistol classes because these are the classes subject to geometry exclusion.

Each of the seven detector configurations is trained independently in all seven folds. The images excluded in each fold are used only for evaluation, with single-object and multi-object performance assessed separately using $F_{2}$ and ${\mathrm{mAP}}_{0.5}$.

For the single-object evaluation, $F_{2}$ and AP are computed separately for the held-out knife and pistol images and averaged to obtain each fold score. The reported single-object $F_{2}$ and ${\mathrm{mAP}}_{0.5}$ are the arithmetic means across the seven folds.

For the multi-object evaluation, predictions and ground-truth boxes from the excluded scenes are pooled within each fold. Class-specific metrics for knives and pistols are macro-averaged to obtain the fold score; the square-plate class is excluded from these reported averages. The reported multi-object $F_{2}$ and ${\mathrm{mAP}}_{0.5}$ are again averaged across the seven folds.

Between-fold variability is summarized across the seven held-out geometry pairs, rather than their transformed image variants. Because the training subsets overlap and only one random seed is used, the fold scores do not represent independent repetitions of the training experiment.

Architecture selection considers single-object geometry generalization, multi-object performance, and inference time separately; these quantities are not combined into a single score.

### 5.2. Final Model and Independent Multi-Object Evaluation

After architecture selection, a new instance of the selected architecture is trained as the final detector using the same initialization and training settings. The complete 412-image synthetic development pool is repartitioned using the 70:15:15 protocol described in Section 4.3. The same training settings are retained, including augmentation restricted to the training subset and checkpoint selection based on validation loss.

Final quantitative performance is evaluated on a separate set of 14 multi-object scenes that remain outside the development pool and do not contribute to architecture selection, training, validation, or checkpoint selection. These scenes include both previously represented and unseen target geometries. They are generated as joint electromagnetic acquisitions following the procedure described in Section 4.2 and are evaluated using the metrics defined in Section 4.4.

### 5.3. Experimental Radar Systems

The final detector is evaluated on measured radar images acquired with three radar systems. The first is the reference D-band radar described in Section 2, whose acquisition configuration is represented in the synthetic training data. Two additional radar systems are used only for measured evaluation.

All three systems follow the same acquisition principle: a linear Tx/Rx array provides the horizontal aperture, while mechanical translation along the *z*-axis synthesizes the vertical aperture. The additional systems preserve this overall acquisition scheme while changing the number, spacing, and distribution of array elements and, for the K-band system, the operating frequency band.

The later-generation D-band array operates over approximately the same frequency range as the reference radar but uses a different array configuration, with 15 transmitters and 15 receivers. The Tx and Rx elements form two parallel linear subarrays with spacings of 50.07mm and 70.76mm, respectively.

The K-band system operates from 23 to 26.3GHz and comprises 11 transmitters and 11 receivers. The Tx and Rx elements again form two parallel linear subarrays, with spacings of 63mm and 100mm, respectively.

The three measurement systems are summarized in Table 3.

Measured data are reconstructed using the acquisition parameters of each radar and converted to the common detector-input representation defined in Section 4.1.

Two scenes are evaluated for each radar system: one containing two knives and one containing a pistol, giving six measured scenes in total. The reference D-band case tests simulation-to-measurement transfer for the modeled acquisition, while the later-generation D-band and K-band systems extend the evaluation to different acquisition configurations.

**Figure 6:**
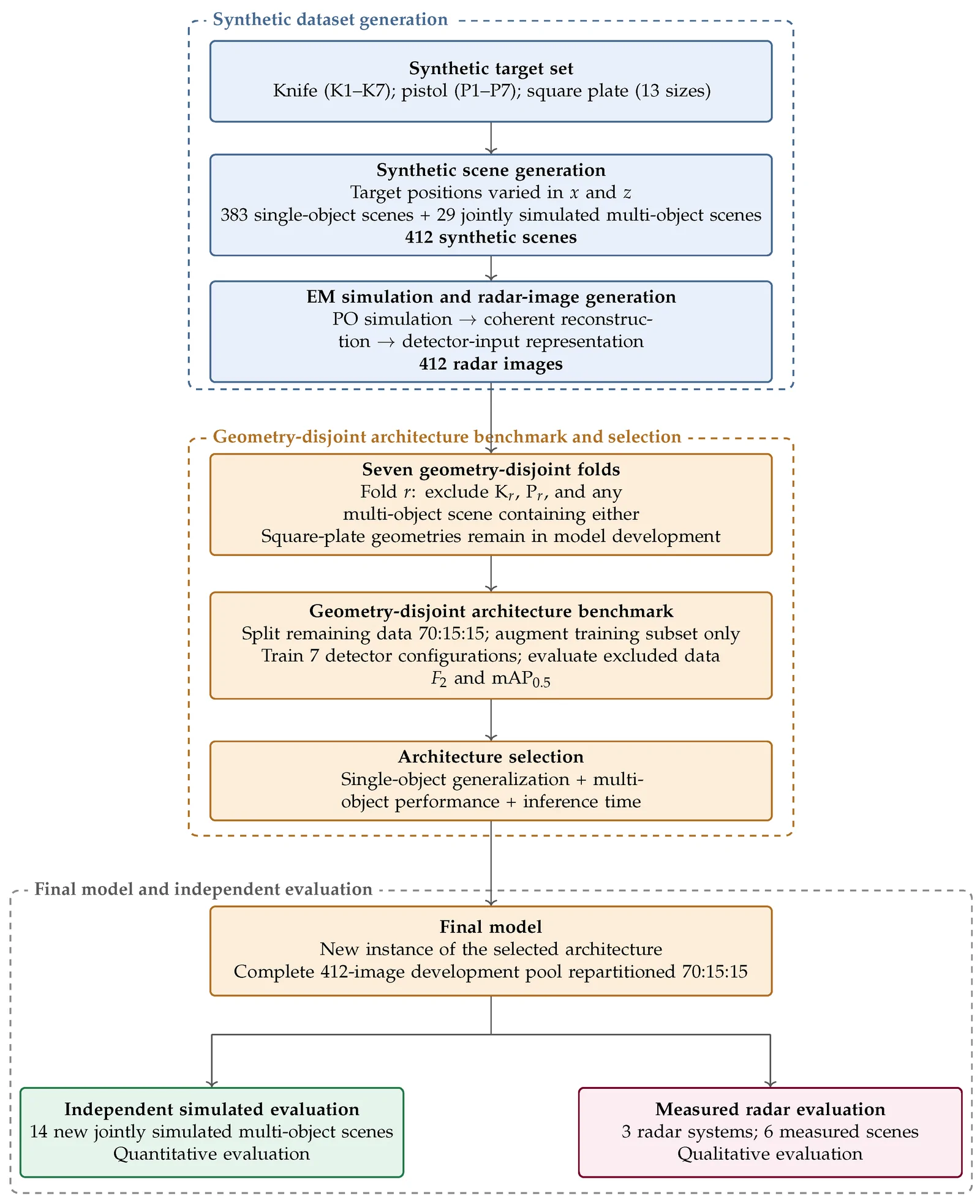
Detector-development and evaluation workflow. Architecture selection uses the geometry-disjoint benchmark, after which a new final detector is trained following a new partition of the complete synthetic development pool and evaluated on independent simulated and measured data.

Given the limited number of measured scenes, these results are interpreted qualitatively rather than as a statistical estimate of cross-system performance. Figure 6 summarizes the complete detector-development and evaluation workflow.

## 6. Results and Discussion

Results are organized in three stages. The geometry-disjoint benchmark evaluates generalization to target geometries excluded from model development and supports architecture selection. The selected model is then evaluated on new jointly simulated multi-object scenes for independent quantitative assessment within the modeled radar domain, and finally on measured data from the reference D-band radar and two additional systems to examine simulation-to-measurement transfer under different acquisition conditions.

### 6.1. Geometry-Disjoint Benchmark and Architecture Selection

Table 4 summarizes the geometry-disjoint benchmark across the seven folds, including single-object and multi-object performance and detector inference time.

Single-object performance is relatively similar among the strongest one-stage detectors. YOLOX-Tiny obtains the highest mean values, with $F_{2}=0.89$ and ${\mathrm{mAP}}_{0.5}=0.88$. The corresponding sample standard deviations across the seven held-out geometry folds are 0.056 and 0.064, respectively.

Differences between architectures become more pronounced in the multi-object evaluation. YOLOX-Tiny again obtains the highest mean values ($F_{2}=0.82$, ${\mathrm{mAP}}_{0.5}=0.79$), whereas several architectures that perform similarly on isolated targets deteriorate more substantially in jointly reconstructed scenes. YOLOv4-Tiny illustrates this behavior: its single-object results are close to those of YOLOX-Tiny, but its multi-object $F_{2}$ decreases from 0.88 to 0.73. Thus, performance on a held-out geometry in isolation does not necessarily translate to comparable performance in multi-object scenes.

Inference time provides a separate computational criterion. SSD-ResNet50 is the fastest detector at 5.86ms, while YOLOX-Tiny requires 10.97ms per input. YOLOX-Tiny nevertheless remains within the timing range of the one-stage detectors while achieving the highest mean detection performance in both geometry-disjoint evaluations.

Considering geometry-disjoint single-object performance, multi-object performance, and inference time, YOLOX-Tiny is selected for the final model. This selection reflects the overall balance of these three criteria rather than the small differences observed in individual performance metrics.

### 6.2. Independent Multi-Object Evaluation

After architecture selection, the final YOLOX-Tiny detector is evaluated on the 14 independent multi-object scenes kept outside model development. It achieves $F_{2}=0.83$ and ${\mathrm{mAP}}_{0.5}=0.79$ when averaged across all three classes, providing an independent quantitative assessment within the modeled radar domain.

Square plates achieve higher detection scores ($F_{2}$ = 0.94, AP = 0.97) than knives and pistols. When the macro-average is restricted to these two classes, $F_{2}$ decreases from 0.83 to 0.77 and mAP from 0.79 to 0.71.

**Figure 7:**
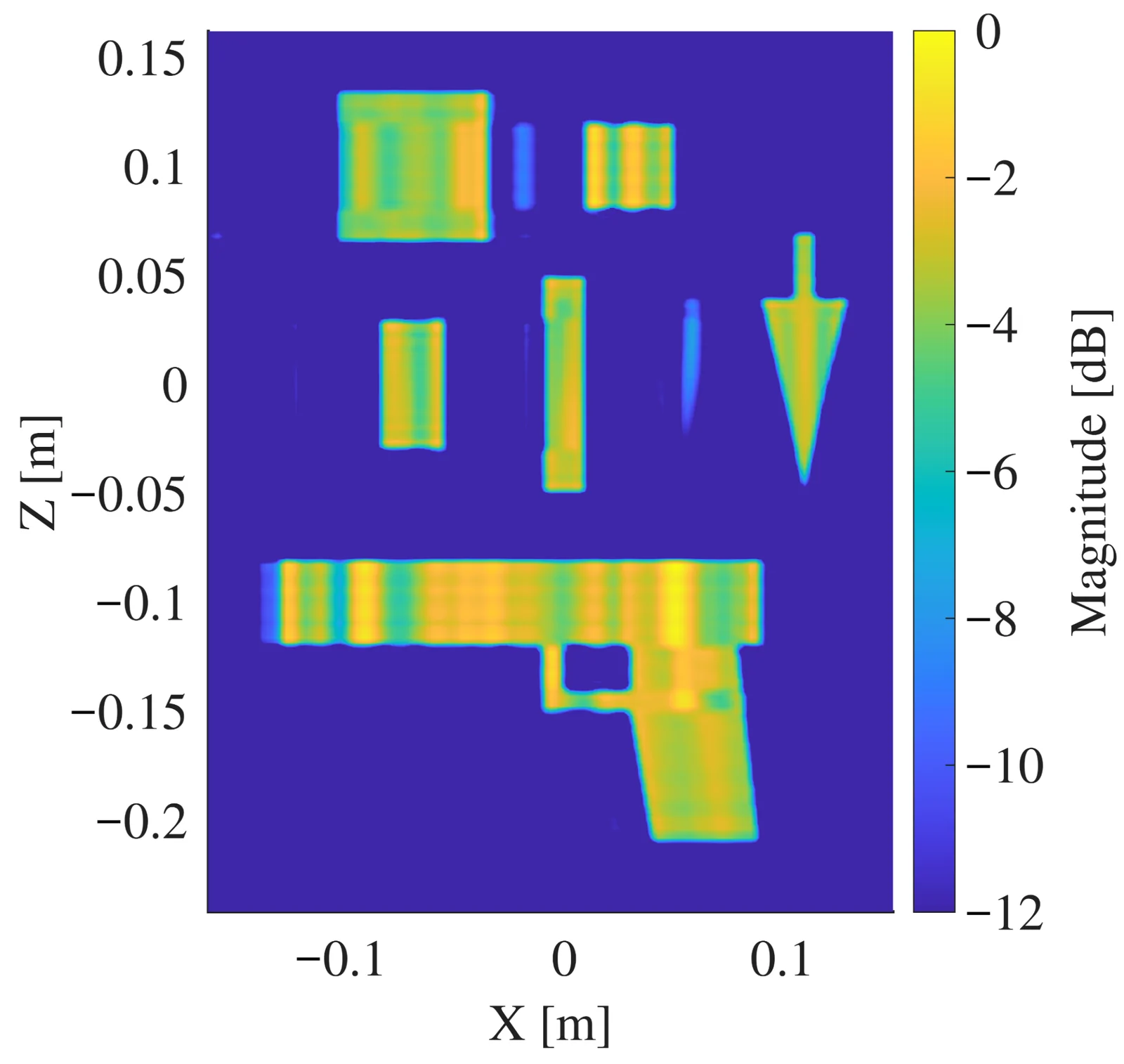
Reconstructed radar image of the diagnostic jointly simulated multi-object scene.

Aggregate metrics do not distinguish between errors caused by the detector itself and those associated with the radar-image formation process. To examine these mechanisms, we consider a diagnostic jointly simulated scene containing one pistol, one knife, two square plates, and two rectangular metallic distractors outside the trained label set. The corresponding reconstructed radar image is shown in Figure 7.

**Figure 8:**
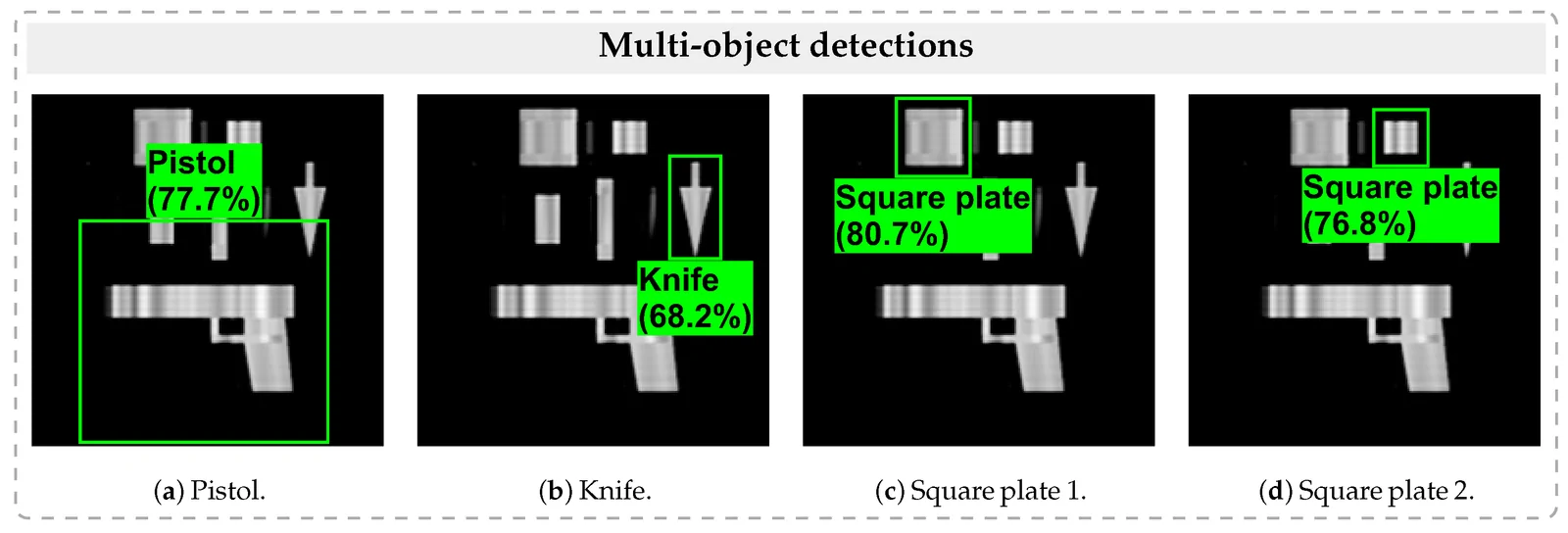
YOLOX-Tiny detections in the diagnostic jointly simulated multi-object scene: (**a**) pistol, (**b**) knife, and (**c**,**d**) the two square plates.

YOLOX-Tiny correctly localizes and classifies all four labeled targets in this scene: the pistol, the knife, and both square plates, as shown in Figure 8.

**Figure 9:**
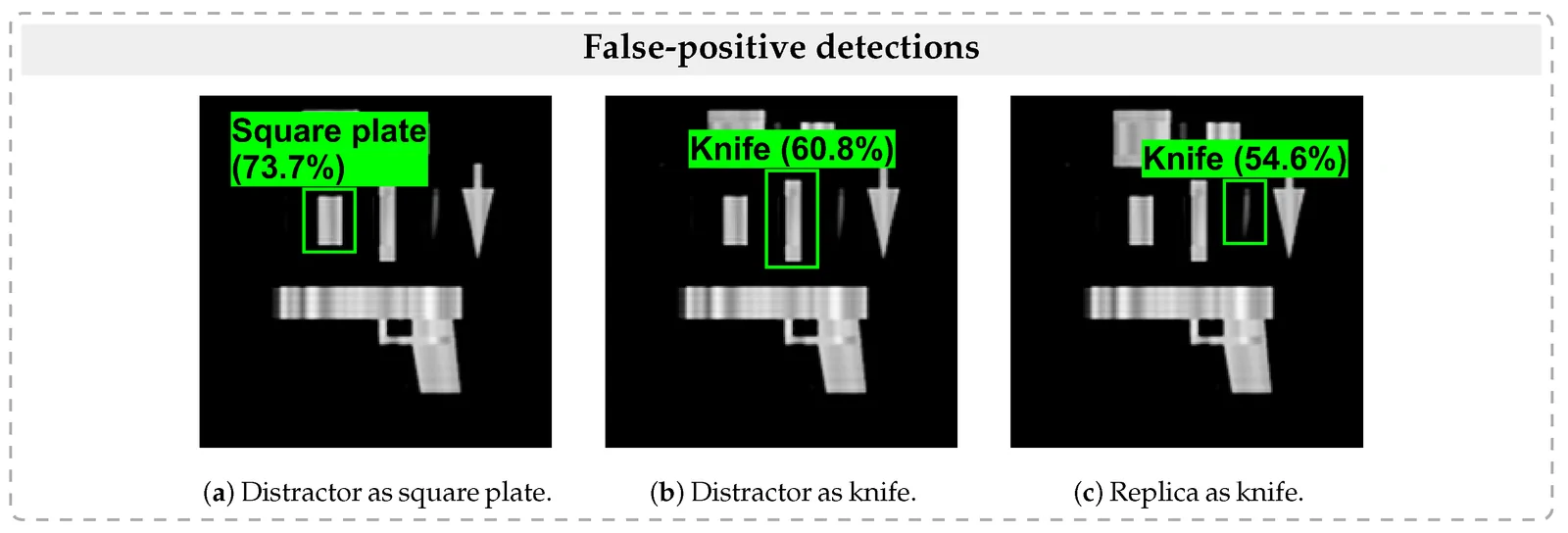
False-positive YOLOX-Tiny detections in the diagnostic jointly simulated multi-object scene. Panels (**a**) and (**b**) show out-of-set metallic distractors classified as square plate and knife, respectively. Panel (**c**) shows a knife detection associated with a radar-image replica rather than a physical target.

However, the same scene also reveals three false-positive detections, as shown in Figure 9. These arise from two distinct mechanisms: responses from physical objects outside the trained label set and a replica introduced by radar-image formation.

Figure 9a,b shows that the two rectangular distractors also generate responses that exceed the detection threshold and are assigned to the square-plate and knife classes, respectively. This behavior is consistent with the closed-set formulation of the detector: once an out-of-set response triggers a detection, classification is restricted to the learned classes. Its radar-image morphology is therefore matched to the available learned representations, and the highest-scoring class is returned, even though the physical object does not belong to that class.

A different mechanism accounts for the additional detection in Figure 9c. No physical object is present at this location. Instead, a replica appears in the reconstructed radar image. Because this response is spatially localized and resembles the morphology of a learned target, the detector assigns it to the knife class, resulting in a false positive.

The diagnostic scene consequently separates two qualitatively different error sources: classification of physical objects outside the trained label set and detections induced by the radar-image formation process.

### 6.3. Evaluation on Measured Radar Systems

The six measured scenes are used as diagnostic cases to examine how target-related characteristics learned from simulation transfer to the reference D-band radar and the two additional systems.

These measurements are not intended to provide a statistical estimate of cross-system detection performance. Instead, they are used to examine how changes in the reconstructed radar response affect detector behavior.

The confidence scores require care in interpretation. For each detection, the MATLAB YOLOX implementation used here produces an objectness score, which reflects how strongly a region appears target-like, and a score for its assigned class. Their product is the reported confidence. A substantial class score therefore does not by itself establish that the region corresponds to a physical target or that its assigned class is correct.

Accordingly, confidence is not interpreted as a direct measure of similarity between radar systems. Changes in frequency or array geometry can alter the reconstructed target morphology in different ways, so confidence is considered together with the radar response rather than used to rank cross-system transfer.

#### 6.3.1. Reference D-Band Radar: Acquisition-Matched Measurement

**Figure 10:**
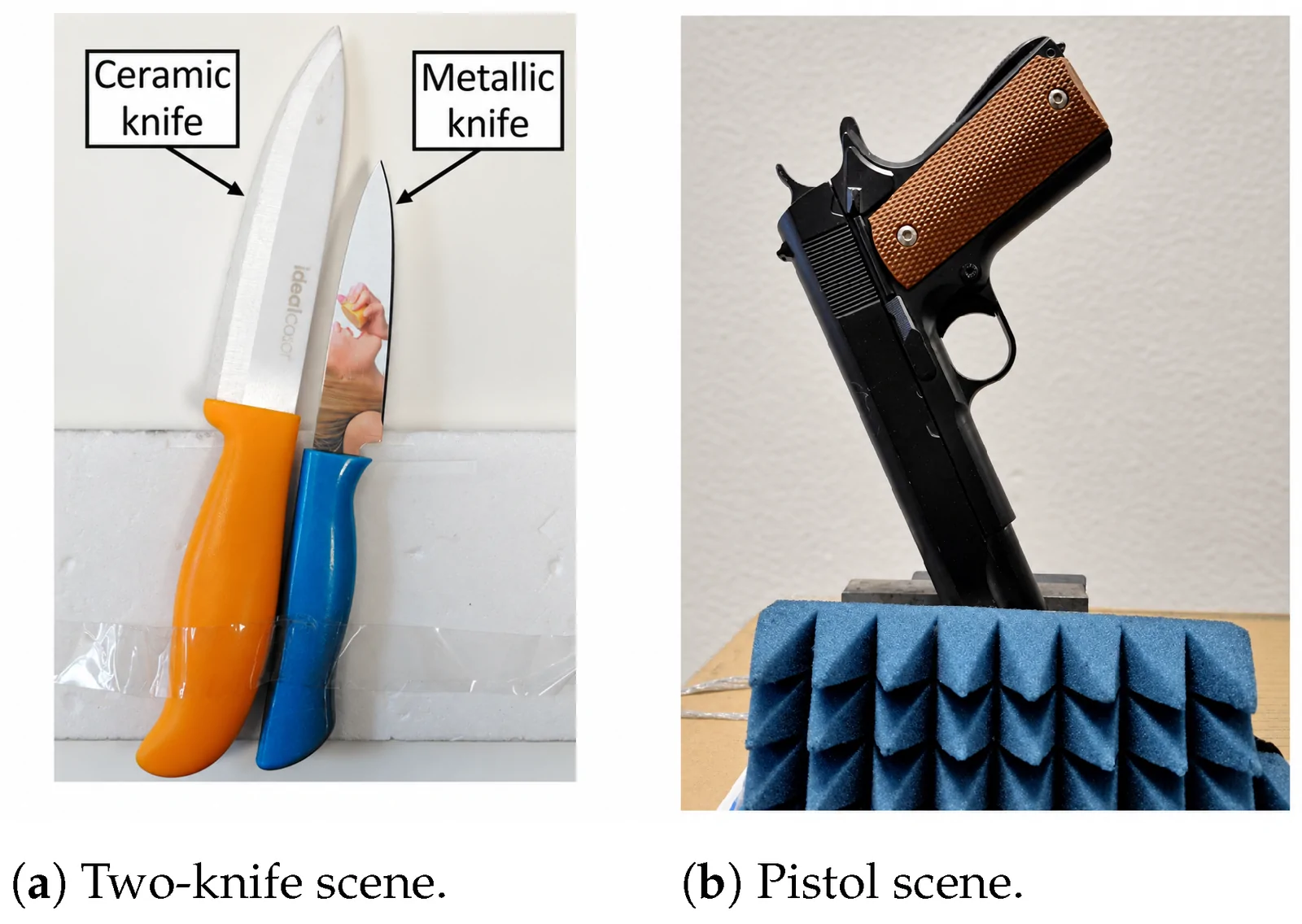
Measurement setups for the reference D-band radar: (**a**) two-knife scene and (**b**) pistol scene.

**Figure 11:**
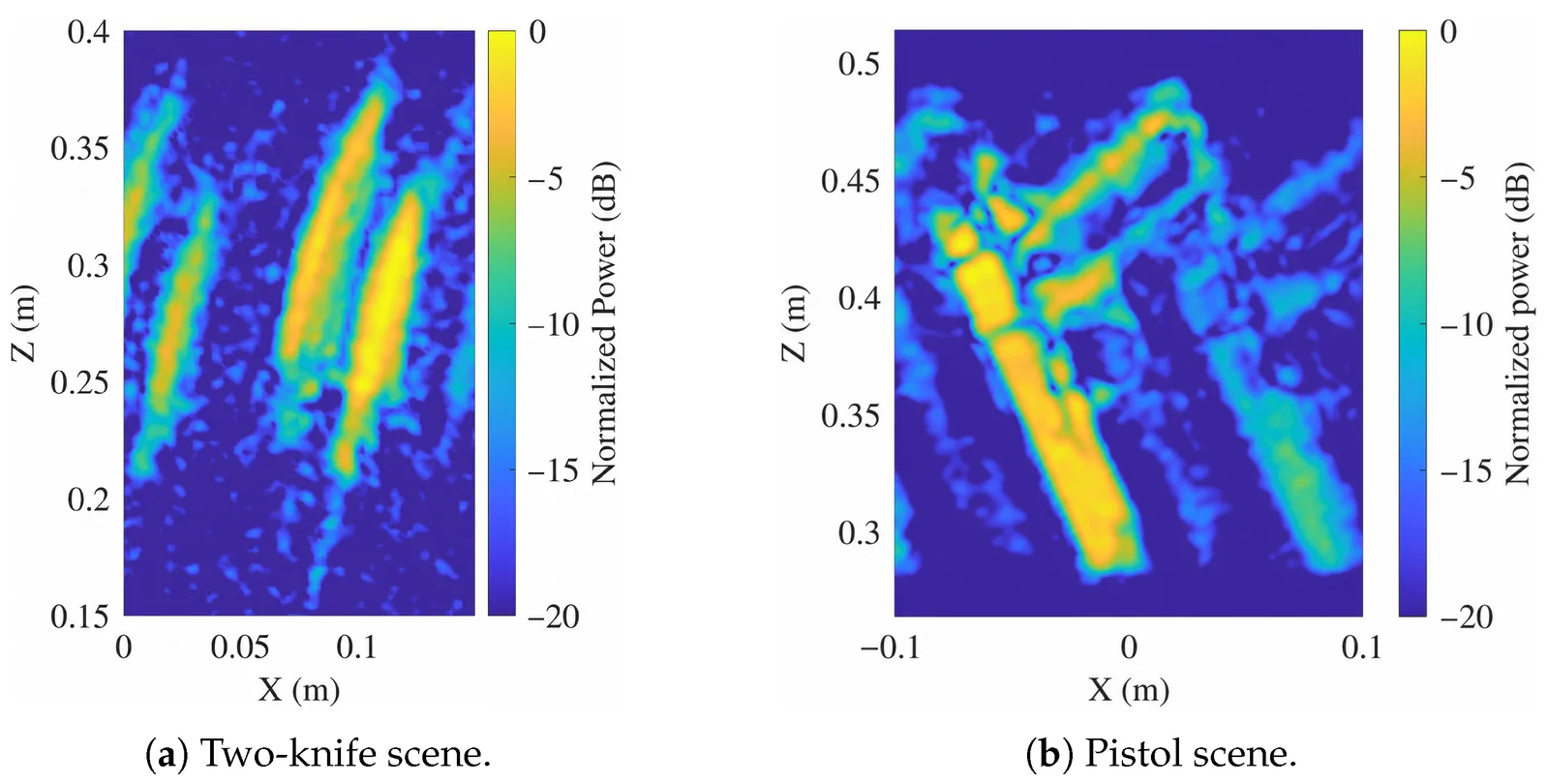
Reconstructed radar images for the reference D-band measurements: (**a**) two-knife scene and (**b**) pistol scene.

The reference D-band measurements provide the acquisition-matched case, since the same radar configuration is represented in the synthetic training data. Two scenes are considered: two closely spaced knives and a single pistol, shown in Figure 10. Their reconstructed images in Figure 11 show the physical targets together with structured replica responses.

**Figure 12:**
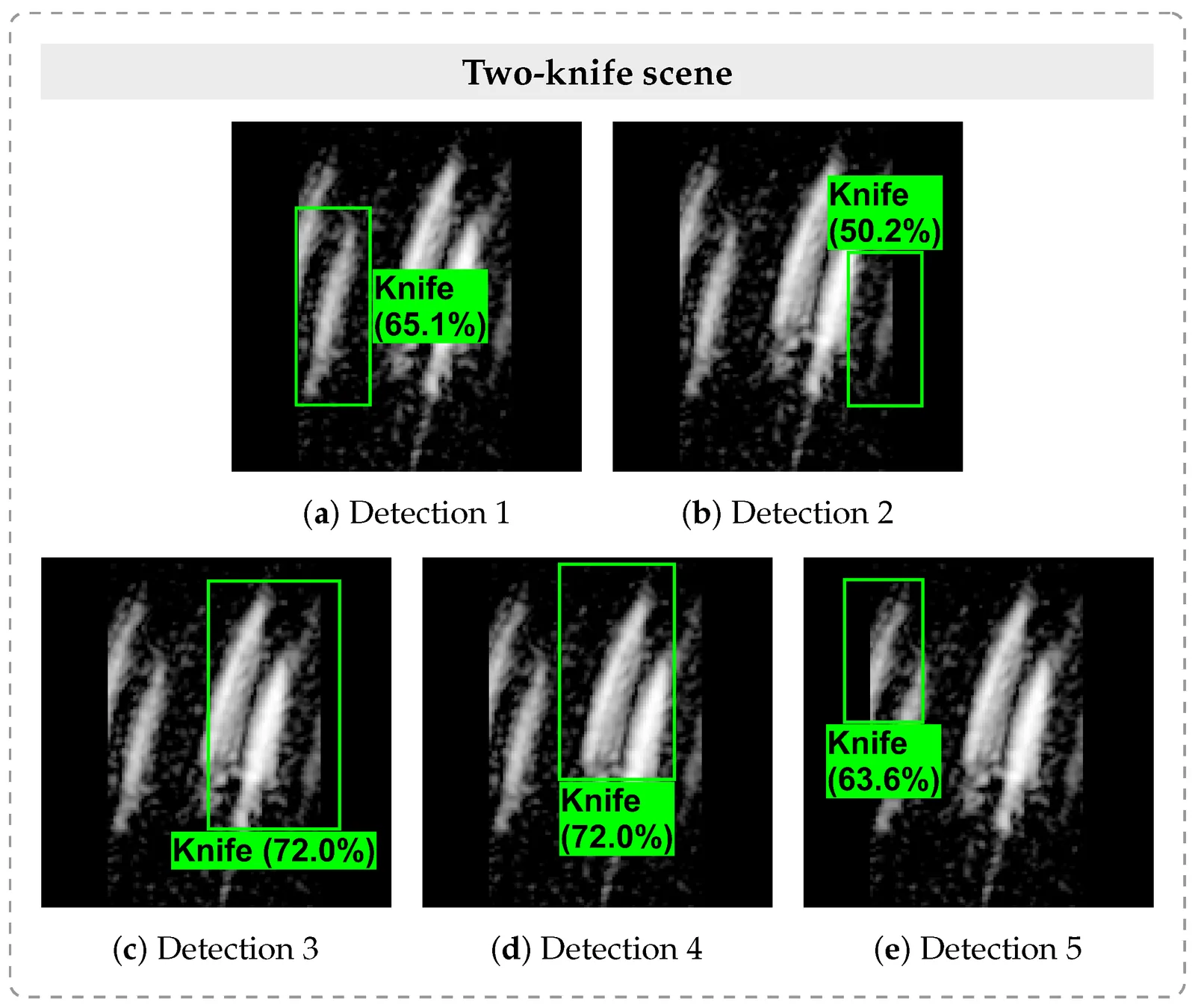
YOLOX-Tiny detections for the reference D-band two-knife measurement scene. All detections at or above the 0.5 confidence threshold are shown.

The two-knife scene contains two closely spaced physical targets and nearby radar replicas. The five detections shown in Figure 12 are all assigned to the knife class. Detection 3 aligns with a physical knife response, while the additional boxes cover elongated responses produced by the targets and their replicas. The scene contains only two physical knives. Therefore, although YOLOX returns five knife-labelled detections, these outputs do not correspond one-to-one to physical targets: some are associated with the physical target responses, whereas others are triggered by nearby replica responses.

**Figure 13:**
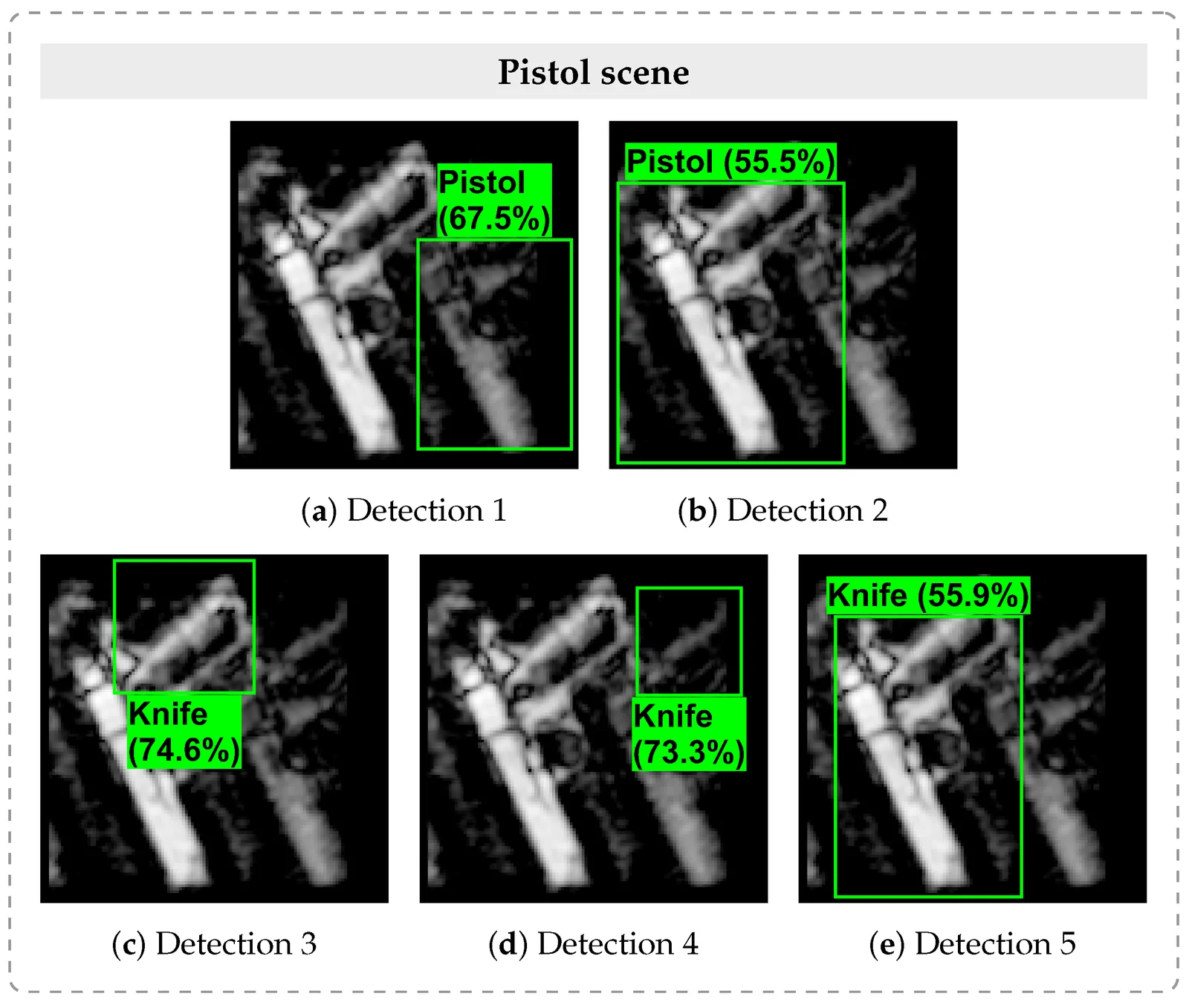
YOLOX-Tiny detections for the reference D-band pistol measurement scene. All detections at or above the 0.5 confidence threshold are shown.

The pistol scene, shown in Figure 13, reveals two further effects. Detection 1 encloses a radar replica, whereas detection 2 covers the dominant response of the physical pistol; both are assigned to the pistol class. The replica is smoother and less detailed than the physical response shown in panel (b) of Figure 11. Within that richer response, three elongated regions trigger detections 3–5, all assigned to the knife class. These boxes do not represent physical knives. They show that local structures of the measured pistol can resemble a different learned target class.

Table 5 reports the detection scores, illustrating that high confidence does not necessarily indicate a distinct physical target. In the two-knife scene, detection 3 combines an objectness score of 0.91 with a knife-class score of 0.79, yielding a final confidence of 0.72. Other detections associated with radar replicas also receive substantial knife-class scores. In the pistol scene, the physical target receives a slightly higher pistol-class score than its replica (0.77 vs. 0.75), but a lower final confidence (0.55 vs. 0.67). Similarly, three incorrect knife detections within the pistol response achieve high class scores (0.77–0.80), indicating that different features of a single target can trigger multiple detections. These observations highlight the distinction between recognizing target-like features and establishing a one-to-one correspondence between detections and physical targets.

#### 6.3.2. Later-Generation D-Band Array: Array-Geometry Shift

**Figure 14:**
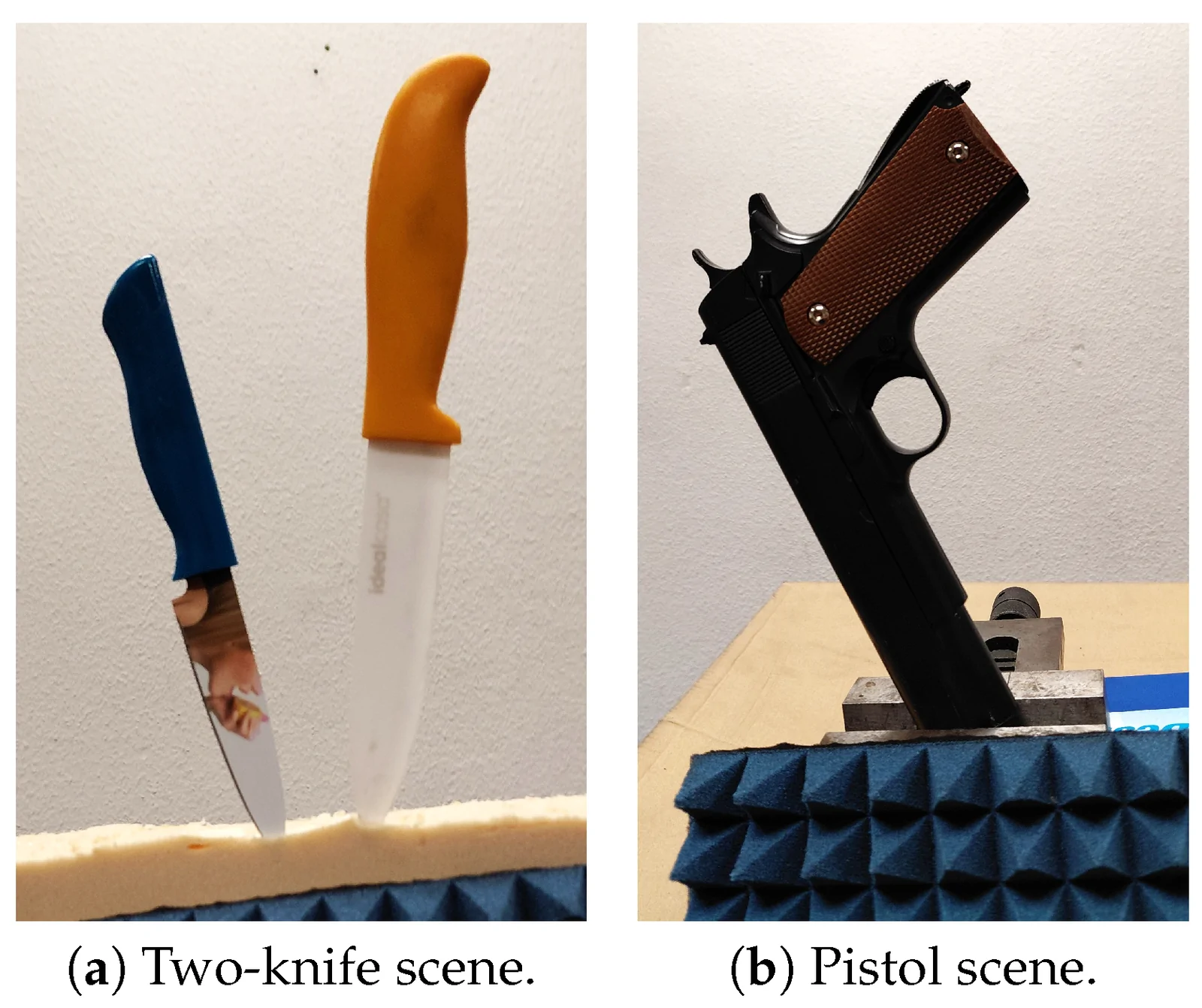
Physical target arrangements used for the measurements acquired with the additional radar systems. The same two scenes were measured with both the later-generation D-band array and the K-band array: (**a**) two-knife scene and (**b**) pistol scene.

For the additional D-band and K-band measurements, the positions of the two knives were adjusted slightly with respect to the reference D-band experiment to improve their placement and separation during acquisition. The physical target arrangements used with both additional systems are shown in Figure 14. The underlying diagnostic scenario remains the same: two nearby knives are measured jointly and reconstructed within a single radar image. These measurements are therefore interpreted as analogous scenes rather than as spatially registered repetitions of the reference D-band acquisition.

**Figure 15:**
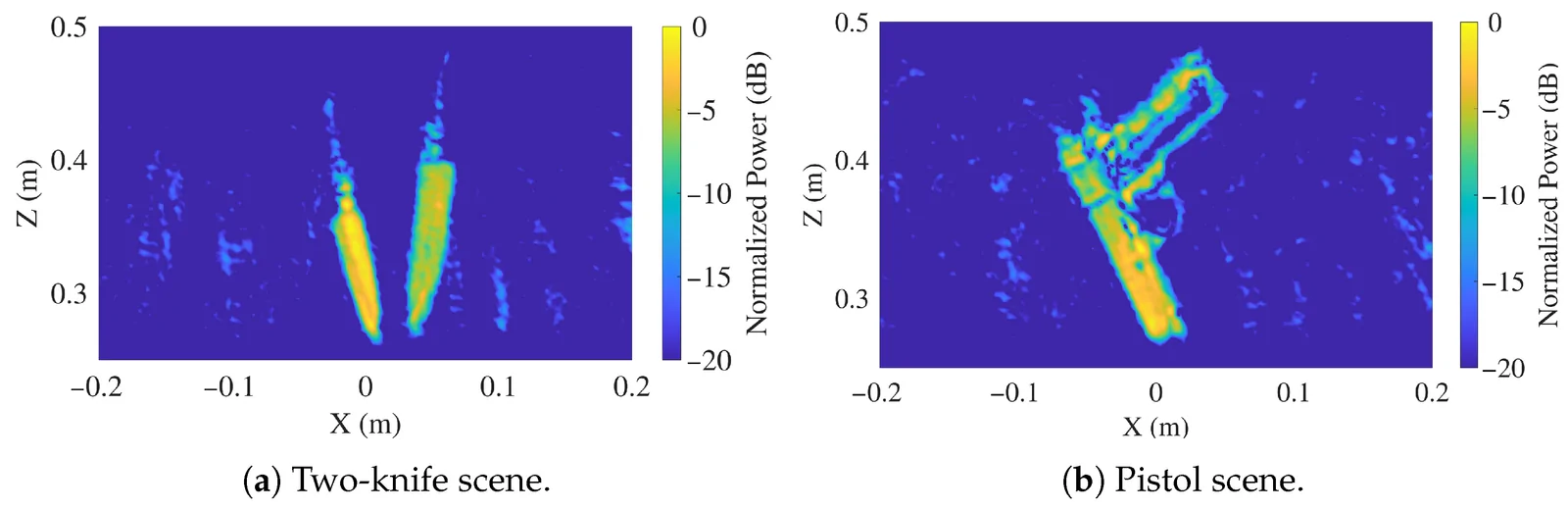
Reconstructed radar images acquired with the later-generation D-band array for the target arrangements shown in Figure 14: (**a**) two-knife scene and (**b**) pistol scene.

**Figure 16:**
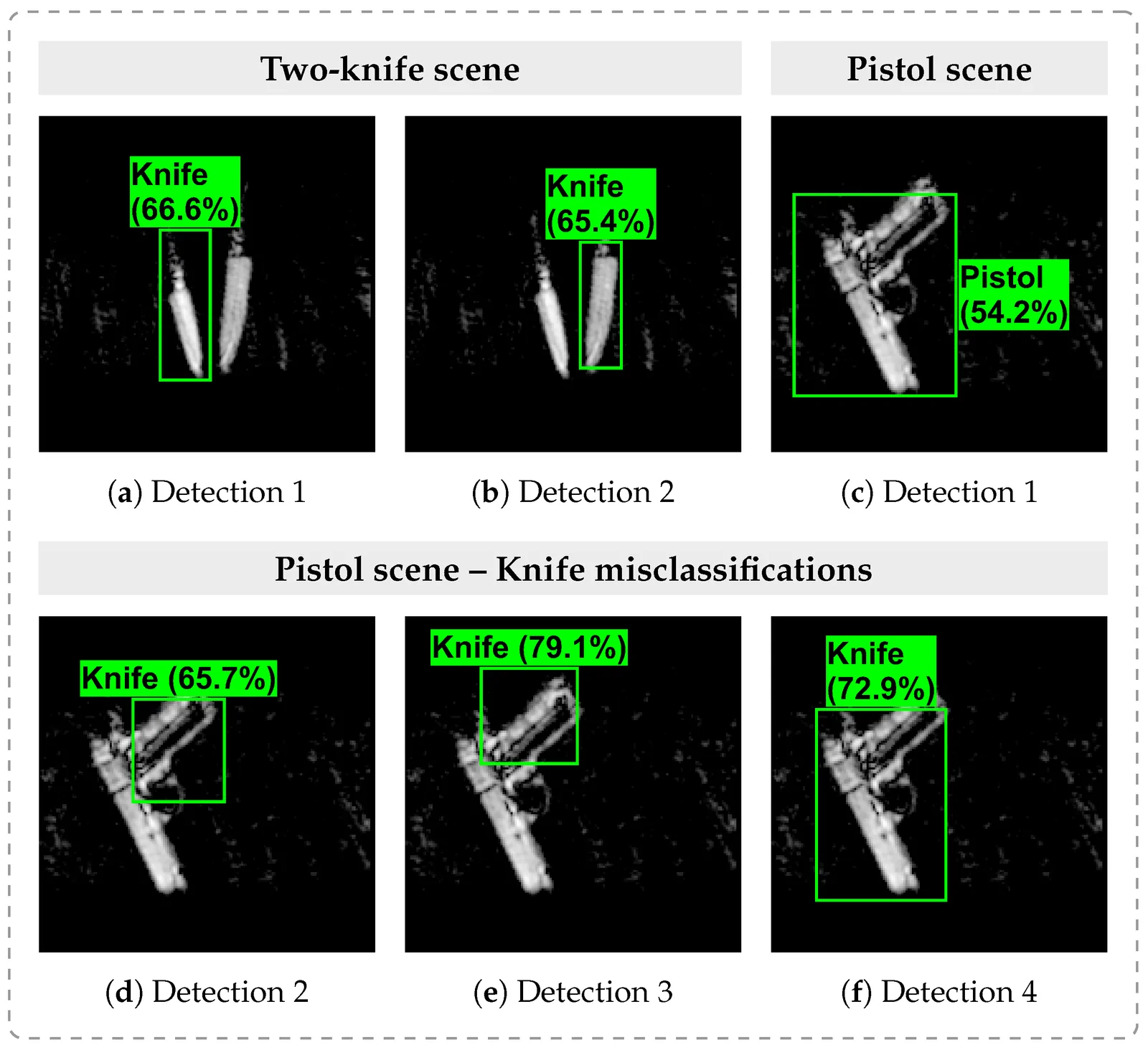
YOLOX-Tiny detections for the later-generation D-band measurements. Panels (**a**,**b**) show the two-knife scene. For the pistol scene, panel (**c**) shows the correctly classified pistol detection, while panels (**d**–**f**) show additional detections incorrectly classified as knives. All detections at or above the 0.5 confidence threshold are shown.

The better-separated knife responses in Figure 15 are reflected in the detector outputs shown in Figure 16. In the two-knife scene, detections (a) and (b) are both assigned to the knife class and align with the two dominant target responses. Unlike the reference D-band measurement, no additional detections are produced by nearby replica structure. The improved separation of the reconstructed responses therefore allows the two physical knives to be resolved as two distinct detector outputs.

The pistol scene in panels (c–f) of Figure 16 shows a different outcome. Detection 1 covers the physical pistol and is assigned to the pistol class. Detections 2–4 are labelled as knives, but their boxes fall within the detailed response of that same pistol rather than on separate radar replicas. The sharp reconstruction in Figure 15 reveals several elongated scattering features across the target. These local features resemble knife-like patterns learned during training, even though the overall target response remains recognizable as a pistol.

Table 6 gives the scores associated with each box. The two physical knives have objectness scores of approximately 0.85 and knife-class scores of 0.78 and 0.77, respectively. Their final confidences are 0.67 and 0.65. These values support the visual result: knife-related features learned with the reference D-band configuration remain recognizable with the additional array, and this scene produces no additional detections above threshold.

For the physical pistol, the model assigns 0.73 to the presence of a target-like response and 0.74 to the pistol class. Their product gives the lower final confidence of 0.54; the displayed score therefore does not indicate an absence of pistol-class evidence. The three additional boxes, however, receive knife-class scores of 0.78–0.81 and final confidences of 0.66–0.79. All three incorrect knife detections have higher final confidence than the correctly labelled pistol; detection 3 has the highest score in the scene. These are substantial responses to distinct parts of the measured target, rather than marginal detections at the confidence threshold.

The difference in replica visibility is primarily explained by the evolution of the MIMO array design. The original D-band array was optimized for a 3 m standoff distance and exhibits prominent PSF replicas close to the main response when operated at approximately 2 m. In contrast, the later-generation array was specifically optimized for this shorter distance, with an increased number of elements and a spatial distribution designed to reduce sidelobe levels and displace the dominant replicas farther from the main response. This substantially extends the region around the physical targets that is less affected by aperture-induced artifacts, explaining why the replicas observed with the original system are no longer apparent in the displayed target region. Importantly, the replicas have not been eliminated; their spatial distribution and relative amplitude have changed. Differences in target placement and other acquisition conditions may also affect their visibility.

#### 6.3.3. Lower-Resolution K-Band System: Cross-Band Evaluation

The K-band measurements introduce the largest acquisition change considered in the experimental evaluation, since both the operating frequency and the spatial resolution differ substantially from those of the D-band system used to generate the synthetic training data. The same physical target arrangements shown in Figure 14 are used for this cross-band evaluation.

**Figure 17:**
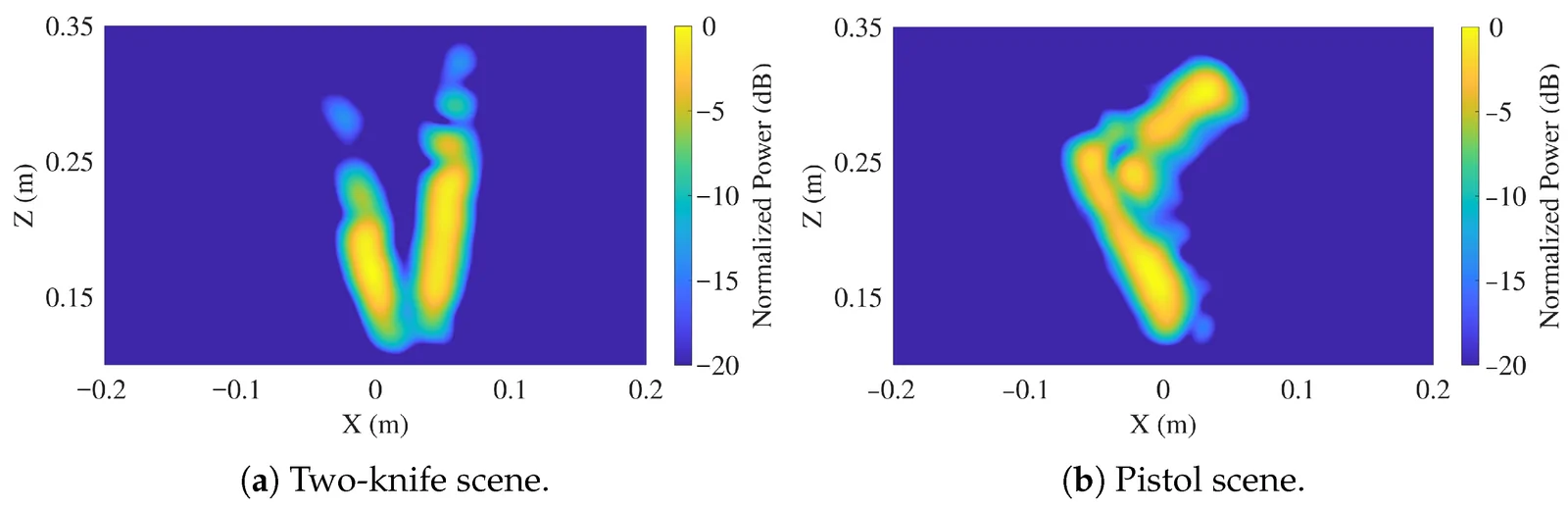
Reconstructed radar images acquired with the additional K-band array for the target arrangements shown in Figure 14: (**a**) two-knife scene and (**b**) pistol scene.

Figure 17 shows a much coarser representation of the targets than the D-band reconstructions. In the two-knife scene, the two physical targets remain clearly separated, but each knife appears as a broad elongated response with little internal detail. The pistol undergoes an even stronger simplification: instead of the rich internal structure observed at D-band, it appears mainly as a smooth, continuous high-energy shape.

The K-band reconstructions contain less visible internal detail than the D-band images while retaining recognizable coarse target shapes. Their simpler appearance is qualitatively closer to that of some synthetic training examples. However, frequency, aperture, bandwidth, and resolution differ between the systems, so these measurements do not isolate the role of resolution in either the response morphology or the detector output.

**Figure 18:**
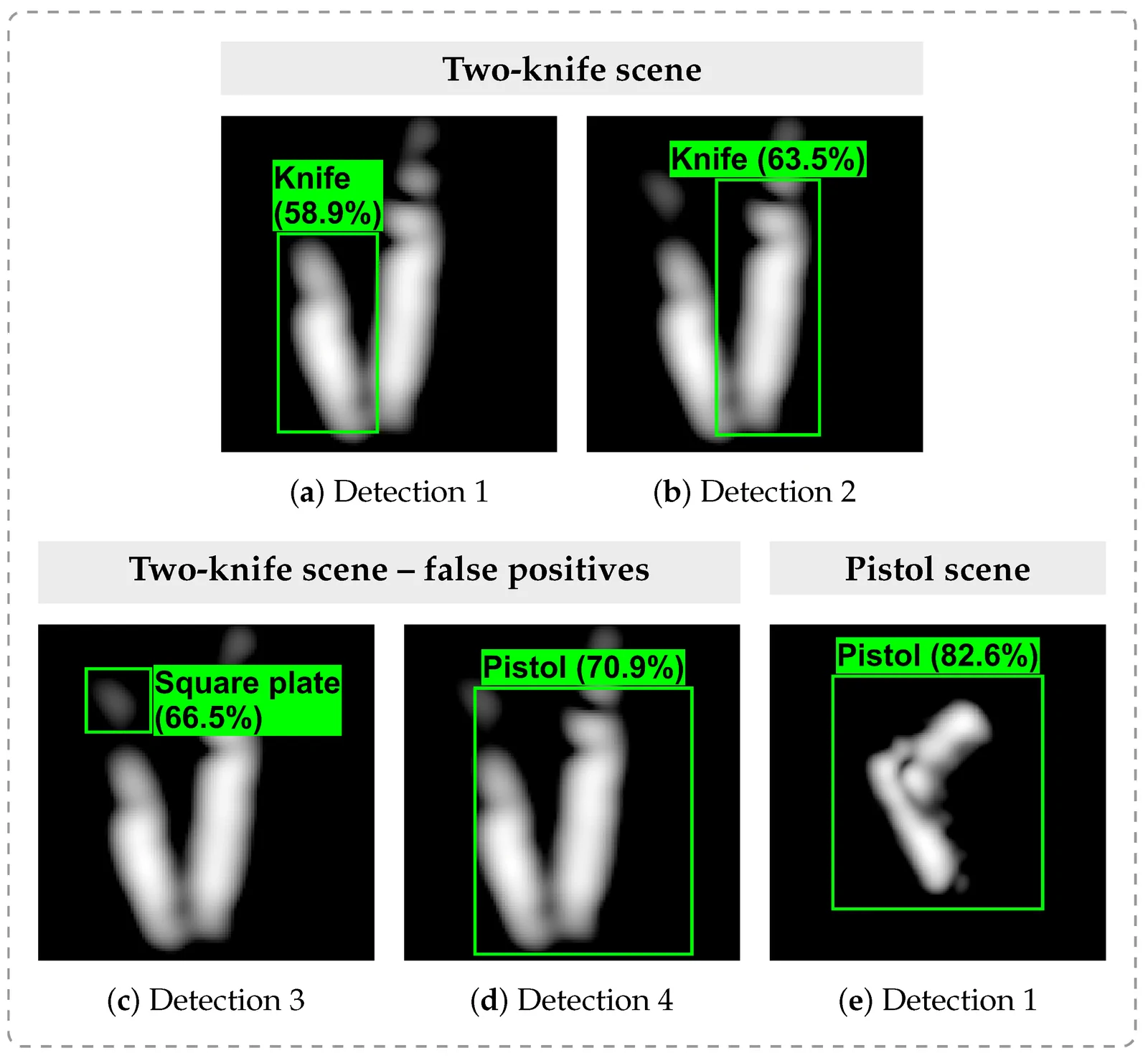
YOLOX-Tiny detections for the measured K-band scenes. Panels (**a**,**b**) show the two correct knife detections in the two-knife scene. Panels (**c**,**d**) show additional detections from the same two-knife scene that are assigned to incorrect classes, whereas panel (**e**) shows the detection obtained for the pistol scene. All detections at or above the 0.5 confidence threshold are shown.

In the two-knife scene, detections 1 and 2 in panels (a) and (b) of Figure 18 correspond to the two physical knives and are assigned to the knife class. The same scene also produces two incorrect assignments. Detection 3 in panel (c) labels a small isolated response as a square plate, while detection 4 in panel (d) labels an elongated response associated with a physical knife as a pistol. Neither box indicates an additional physical target of its assigned class. In the pistol scene, detection 1 in panel (e) covers the physical pistol, assigns it to the correct class, and is the only detection above threshold. Table 7 reports the scores behind these results.

The two physical knives receive knife-class scores of 0.71 and 0.77, indicating that features learned from simulated D-band responses remain recognizable in this measured K-band scene. However, the incorrect square-plate and pistol assignments have final confidences of 0.67 and 0.71, both higher than those of the correctly labelled knives. Their class scores are also substantial, at approximately 0.76.

The physical pistol receives an objectness score of 0.96 and a pistol-class score of 0.86, giving a final confidence of 0.83. Its correct detection without additional boxes above threshold contrasts with the class ambiguity observed in the two-knife scene. Together, these measurements provide qualitative evidence that some detection features learned from simulated D-band responses remain informative in K-band images. The two incorrect class assignments, however, preclude any claim of unambiguous cross-band recognition.

### 6.4. Discussion

The geometry-disjoint benchmark supports generalization to unseen knife and pistol geometries within the modeled D-band configuration. YOLOX-Tiny provides the most favorable observed balance of detection performance and inference cost, but seven folds with overlapping training sets and a single training seed do not establish statistical superiority. Moreover, the 216 image-domain variants per test image do not represent additional independent electromagnetic acquisitions.

The measured results complement previous studies of simulation-to-measurement transfer [11,12] by illustrating how radar-image formation affects detector outputs. Replicas can trigger detections without corresponding physical objects, while distinct scattering features within one target can be classified as separate objects. The later-generation D-band measurements illustrate this distinction: the two-knife scene produces no replica-related detections above threshold, whereas the pistol scene still produces three additional knife detections within the response of a single physical target. Because detections associated with different scattering features of the same target need not overlap, nonmaximum suppression alone cannot reliably associate them with a single physical object. Target-level association could help address this limitation.

The K-band measurements retain correct detections of the physical targets but also produce additional false detections assigned to other classes. Since frequency, bandwidth, and aperture geometry change together, the contribution of resolution to these errors cannot be isolated. The limited measured dataset supports qualitative observations, not quantitative claims of cross-system performance.

For the electrically large, predominantly planar metallic targets considered in this study, the physical-optics (PO) approximation provides a physically well-founded and computationally efficient approach to generating synthetic radar responses. By modeling the currents induced on illuminated surfaces and preserving coherent near-field bistatic propagation, PO captures important scattering characteristics that contribute to the spatial structure of reconstructed radar images. For detector training, the relevant requirement is to preserve sufficiently representative and discriminative radar features, rather than to reproduce every electromagnetic scattering contribution. Although multiple scattering and edge diffraction are not explicitly modeled, their significance depends on target geometry and observation conditions. Multiple interactions between surfaces and diffraction from sharp contours, including those of otherwise planar targets, may introduce additional contributions to the scattered field. These contributions can modify the complex radar responses and the morphology of the reconstructed images, potentially increasing discrepancies between simulation and measurement. Nevertheless, the geometry-disjoint evaluation demonstrates that PO-based synthetic data support generalization to unseen target geometries within the modeled configuration, while the measured scenes provide qualitative evidence that learned features remain informative under different acquisition conditions. Together, these findings support the adequacy of PO for the synthetic training and detection task investigated in this work, without constituting a direct quantitative validation of its electromagnetic accuracy.

The single-object subset is class-imbalanced, comprising 215 knife, 77 pistol, and 91 square-plate images. Although knives and pistols are each represented by seven distinct target geometries, their unequal sample counts may influence class representation during training. No class weighting or class-balanced sampling was applied, and online image-domain augmentation was intended to increase variability and detector robustness rather than to equalize class frequencies.

The class-specific results provide further insight into detector performance under this training distribution. In the geometry-disjoint benchmark, the less-represented pistol class achieves higher mean $F_{2}$ and AP scores at an IoU threshold of 0.5 than the knife class across all seven evaluated architectures, in both single-object and multi-object scenes. For YOLOX-Tiny, the single-object $F_{2}$ scores are 0.94 for pistols and 0.84 for knives, with corresponding AP values of 0.94 and 0.82. The same trend is observed in the multi-object evaluation, with $F_{2}$ scores of 0.90 and 0.75, respectively. In contrast, the separately trained final detector shows slightly lower performance for pistols ($F_{2}$ = 0.76, AP = 0.69) than for knives ($F_{2}$ = 0.78, AP = 0.73) in the independent multi-object evaluation. These observations do not indicate a consistent performance disadvantage for the less-represented class across the evaluations conducted. However, differences in target scattering characteristics, geometric complexity, scene composition, and evaluation conditions may also influence detection performance. Consequently, the effect of class imbalance cannot be isolated from these results. The geometry-disjoint metrics are macro-averaged over knives and pistols, ensuring equal contributions to the aggregate evaluation regardless of sample frequency, although this does not compensate for potential effects of imbalance during training.

The synthetic dataset represents a limited range of physical responses, primarily from simplified planar PEC targets modeled with PO. Future work could examine class-balanced training and incorporate greater diversity in target geometries, materials, and poses, including additional scattering mechanisms where relevant. These extensions would provide physical variability that image-domain augmentation alone cannot reproduce.

## 7. Conclusions

This work shows that synthetic electromagnetic responses can provide useful training data beyond the specific target geometries used to generate them. The simulated evaluations support this finding within the modeled D-band configuration, while measurements from three radar systems suggest that some learned features remain recognizable under different acquisition conditions. However, these measurements also reveal that detecting a target-like response does not always mean identifying a distinct physical object.

The next challenge is to move from recognizing radar-image features to reliably identifying physical targets. Progress toward this goal calls for more representative electromagnetic simulations and methods that associate detections with physical objects. Broader experimental validation is needed to establish the extent of generalization across radar systems.

## Figures and Tables

**Table 1:** D-band MIMO radar acquisition parameters reproduced in the EM simulation and used in the experimental measurements. Boldface identifies the three parameter groups.

Parameter	Value
**Radar waveform**	
Radar type	LFMCW D-band MIMO radar
Frequency band	118–133.6 GHz
Bandwidth	15.6GHz
Number of frequency samples	10,000
**Sparse multistatic aperture**	
Number of transmitters	8
Number of receivers	7
Number of bistatic Tx/Rx channels	8×7=56
Tx spacing	7.2cm
Rx spacing	16cm
**Mechanical scan**	
Scan axis	*z*-axis
Vertical scan range	[−0.35,0.35]m
Vertical scan step	5.6mm
Number of scan positions	126

**Table 2:** Composition of the single-object synthetic radar dataset.

Class	Number of Images
Knife	215
Pistol	77
Square plate	91
**Total**	**383**

**Table 3:** Radar systems used for the measured zero-shot evaluation. Only the reference D-band system is represented in the synthetic detector-training data.

System	Frequency Band (GHz)	$N_{\mathrm{Tx}}$	$N_{\mathrm{Rx}}$	$d_{\mathrm{Tx}}$ (mm)	$d_{\mathrm{Rx}}$ (mm)	Nominal Image-Plane Resolution $\delta_{x}\times \delta_{z}$ (mm)
Reference D-band	118–133.6	8	7	72	160	3.07×3.21
Later-generation D-band	118–133.3	15	15	50.07	70.76	2.66×3.21
K-band	23–26.3	11	11	63	100	13.98×16.28

Note: $\delta_{x}$ and $\delta_{z}$ denote the nominal horizontal and vertical cross-range resolutions in the XZ imaging plane at $R_{0}=2\;m$, estimated as $\delta_{x}\approx \lambda_{\min}R_{0}/D_{x}$ and $\delta_{z}\approx \lambda_{\min}R_{0}/D_{z}$, where $\lambda_{\min}$ is the minimum wavelength of the corresponding radar system, and $D_{x}$ and $D_{z}$ are the effective horizontal and vertical apertures, respectively.

**Table 4:** Geometry-disjoint benchmark averaged across the seven folds. Detection metrics are macro-averaged over the knife and pistol classes in both the single-object and multi-object evaluations. The square-plate class remains in the training data and multi-object scenes but is excluded from these averages. The selected architecture is highlighted in green; bold values indicate the best mean value in each column.

Detector	Single-Object		Multi-Object		Inference
	$F_{2}$	${\mathrm{mAP}}_{0.5}$	$F_{2}$	${\mathrm{mAP}}_{0.5}$	$t_{\inf}$ (ms)
**YOLOX-Tiny**	0.89	0.88	0.82	0.79	10.97
YOLOX-S	0.87	0.83	0.76	0.71	11.30
YOLOv4-Tiny	0.88	0.87	0.73	0.74	7.56
SSD-MobileNet	0.84	0.82	0.57	0.58	7.18
SSD-ResNet50	0.83	0.81	0.73	0.71	5.86
Faster R-CNN-ResNet18	0.80	0.76	0.48	0.33	48.56
Faster R-CNN-MobileNet	0.83	0.77	0.49	0.34	22.36

**Table 5:** Objectness, assigned-class score, and final confidence for detections in the measured reference D-band scenes. Green-highlighted rows identify detections that localize a physical target and assign it the correct class.

Detection	Assigned Class	Objectness	Class Score	Confidence
**Two-knife scene**				
Detection 1 (a)	Knife	0.87	0.75	0.65
Detection 2 (b)	Knife	0.72	0.70	0.50
Detection 3 (c)	Knife	0.91	0.79	0.72
Detection 4 (d)	Knife	0.89	0.81	0.72
Detection 5 (e)	Knife	0.93	0.68	0.64
**Pistol scene**				
Detection 1 (a)	Pistol	0.90	0.75	0.67
Detection 2 (b)	Pistol	0.72	0.77	0.55
Detection 3 (c)	Knife	0.96	0.77	0.75
Detection 4 (d)	Knife	0.92	0.80	0.73
Detection 5 (e)	Knife	0.72	0.77	0.56

**Table 6:** Objectness, assigned-class score, and final confidence for detections in the measured later-generation D-band scenes. Green-highlighted rows identify detections that localize a physical target and assign it the correct class.

Detection	Assigned Class	Objectness	Class Score	Confidence
**Two-knife scene**				
Detection 1 (a)	Knife	0.85	0.78	0.67
Detection 2 (b)	Knife	0.85	0.77	0.65
**Pistol scene**				
Detection 1 (c)	Pistol	0.73	0.74	0.54
Detection 2 (d)	Knife	0.84	0.78	0.66
Detection 3 (e)	Knife	0.98	0.81	0.79
Detection 4 (f)	Knife	0.92	0.80	0.73

**Table 7:** Objectness, assigned-class score, and final confidence for detections in the measured K-band scenes. Green-highlighted rows identify detections that localize a physical target and assign it the correct class.

Detection	Assigned Class	Objectness	Class Score	Confidence
**Two-knife scene**				
Detection 1 (a)	Knife	0.83	0.71	0.59
Detection 2 (b)	Knife	0.82	0.77	0.63
Detection 3 (c)	Square	0.88	0.76	0.67
Detection 4 (d)	Pistol	0.93	0.76	0.71
**Pistol scene**				
Detection 1 (e)	Pistol	0.96	0.86	0.83

## Data Availability

The raw data supporting the conclusions of this article will be made available by the authors on request.
